# Elastic Ranking-Driven Time Series Feature Optimization for Aeroengine Remaining Useful Life Prediction

**DOI:** 10.3390/e28101067

**Published:** 2026-09-28

**Authors:** Ruihao Xin, Sudan Bai, Jiankang Fan, Cong Gao, Xin Feng

**Affiliations:** 1 School of Electrical and Control Engineering, Jilin University of Chemical Technology, Jilin 132022, China; 2 School of Artificial Intelligence, Jilin University of Chemical Technology, Jilin 132105, China

**Keywords:** hierarchical recurrent neural network, context-gated mechanism, elastic sorting of time series indicators

## Abstract

This paper proposes a hierarchical recurrent neural network framework that leverages a context-gated mechanism and the elastic sorting of time series indicators, addressing the strong dynamic coupling characteristics of aeroengine operational time series data. This framework addresses the coupling of multi-physical field signals and the interleaved cross-scale degradation characteristics of engine time series data. The framework achieves high-precision predictions of residual service life under complex operating conditions by deeply integrating multi-modal feature engineering with hierarchical time series modeling. An elastic ranking algorithm for time series indexes is designed to adaptively fuse multiple sensors’ time domains, frequency domains, and nonlinear characteristics, thereby constructing a dynamic feature space sensitive to load conditions. This approach addresses the limitations of traditional fixed feature combinations in characterizing variable load conditions. Additionally, a multi-scale stack dilation convolution module is introduced to exponentially enlarge the receptive field, allowing for the capture of long-range dependencies in weakly degraded signals. A bidirectional time–frequency converter decomposes the signal into low-frequency trend components and high-frequency anomaly components, enabling coupling characterization through cross-scale feature interactive gating. Furthermore, context-gated cyclic units based on hierarchical cyclic networks are constructed to hierarchically integrate multi-modal features while dynamically adjusting time-dependent weights, thereby enhancing the model’s causal reasoning ability for long sequence degradation patterns. In four typical experiments conducted on the C-MAPSS dataset, the ERCG-HRNN demonstrates significant improvements in RMSE and Score compared to existing methods.

## 1. Introduction

In aviation, an aeroengine is the core component of the aircraft power system [1,2]. Its reliability is directly related to flight safety. Due to long-term operation under extreme conditions of high temperature, high pressure and high speed, the engine inevitably experiences progressive degradation caused by cumulative effects such as material fatigue and thermal damage. Accurately describing the mapping relationship between its degradation trajectory and residual service life (RUL) is the core scientific problem in accurately predicting RUL [3]. The RUL is the core of Predictive Health Management (PHM) technology [4,5]. As a cutting-edge technology that ensures high-end equipment’s safe and reliable operation, PHM provides decision support for equipment maintenance through real-time monitoring and intelligent analysis. Accurate prediction of aeroengine RUL is significant for early failure warning, accident prevention and scientific maintenance-strategy formulation [6].

Currently, RUL prediction methods are divided into physics-based degradation models and data-driven models [7,8,9]. Physically degenerate models depend on a deep understanding of the physical evolution of system parameters and their coupling mechanisms, although classical algorithms such as least squares regression and Kalman filter have been developed [10] and applied to aeroengine RUL prediction. However, the complex dynamic response characteristics, coupling effects of multiple physical fields and nonlinear degradation behavior under extreme operating conditions of aero-engine bring significant challenges to constructing a universal high-precision degradation model. In contrast, data-driven methods show unique advantages in RUL prediction of complex systems by mining degradation patterns in monitoring data. They do not need to explicitly establish a physical model. However, they automatically extract the mapping relationship between features and RUL through a machine learning algorithm, especially suitable for engineering practical scenes with intense noise and multivariate coupling.

In recent years, traditional machine learning methods such as Naive Bayes (NB) [11], Support Vector Machine (SVM) [12] and Random Forest (RF) [13] have been widely used in the field of RUL prediction. These methods extract degradation features from multi-source time series monitoring data and establish the mapping relationship between features and RUL. For example, Selina S et al. [14] proposed a Naive Bayes (NB) model for RUL prediction of batteries under different operating conditions; Liu et al. [15] proposed a comprehensive parameter selection method based on information entropy, correlation analysis and oil degradation analysis to establish an RUL prediction model of lubricating oil; Nieto et al. [16] proposed a hybrid model based on PSO-SVM, which combines support vector machine (SVM) and particle swarm optimization (PSO) technology to predict engine RUL. However, aircraft engine operation data has obvious time series characteristics. Its performance degradation is a dynamic process that evolves. Traditional machine learning methods are primarily based on static data assumptions, which cannot fully capture the evolution of data with time, resulting in difficulty in accurately fitting the degradation trend of the engine in practical applications, thus affecting the accuracy and reliability of RUL prediction.

In contrast, deep learning approaches offer new opportunities to address these limitations of traditional machine learning. Deep learning models, such as recurrent neural networks (RNN) [17], their variants (Long Short-Term Memory Networks (LSTM) [18], Gated Recurrent Units (GRU) [19], etc., can naturally handle time-dependent relationships in data due to their powerful sequence modeling capabilities. By calculating time steps, these models could deeply mine the time series pattern of the engine operating data, thereby more accurately depicting the deterioration process of the engine performance over time. Recent Transformer-based models for RUL prediction use self-attention to model long-range dependencies but struggle with fixed sequence segmentation and redundant sensor data sensitivity. GNNs address multi-sensor spatial correlations via static graphs but fail to adapt to dynamic sensor correlation changes during aeroengine degradation. Sayah et al. [20] presented a framework based on deep short- and long-term memory (LSTM) for RUL prediction of aircraft engines; Sheng et al. [21] proposed a new lightweight difference learning mechanism, which uses different weight updating rules to store different trend information without adding any parameters—it is applied to predicting aircraft engine RUL; Liu et al. [22] proposed a new multi-head self-attention entirely convoluted network (MSA-FGN) for predicting the RUL of the turbofan engine; Shuhan et al. [23] proposed a 1D CNN-GRU prediction model for a few samples; Feng et al. [24] proposed a new lightweight framework, AutoFE-Pointer, leveraging an improved softened pointer network for dynamically extracting and weighting features from diverse DNA sequences; Gao et al. [25] proposed an interpretable RUL prediction method (ISTLSTM) based on spatiotemporal features (STFs) for aeroengines under complex operating conditions; Kui et al. [26] proposed a new ensemble method of deep bidirectional recurrent neural networks (DBRNNs) for RUL prediction of aircraft engines; Chengying et al. [27] proposed a new bootstrap ensemble learning convolutional simple cyclic unit (ELCSRU) method for RUL prediction; Tongyang et al. [28] proposed a multi-attention network coupled with adaptive meta-shift learning for RUL prediction; Zheng et al. [29] proposed a causal graph-based spatiotemporal attention network (CaSTAN) for RUL prediction of complex systems; Qi et al. [30] designed a dual frequency domain enhancement network (SDFEPN) to predict aircraft engine RUL through the information criterion (IVC) index and information threshold (CIT) equation; Xingqiu et al. [31] proposed an Integrated Deep Multi-scale Feature Fusion Network (IDMFN) and designed a multi-scale feature extraction block to learn different scale features for aeroengine RUL prediction; Feng et al. [32] proposed a multi-feature fusion method based on an attention mechanism and a data-driven prediction framework that can solve the long-term dependence relationship between time series data and multi-modal feature fusion.

An innovative method is proposed to fuse the feature construction of time series index elastic ranking and multi-scale feature fusion for the aeroengine RUL problem. By analyzing the dynamic characteristics of multi-sensor time series data, an adaptive feature selection and weight assignment mechanism based on an elastic sorting algorithm is constructed: Firstly, key time series indexes reflecting the degradation trend are extracted from engine performance parameters, and adaptive feature extraction under complex operating conditions is realized by dynamically adjusting index weights using the elastic ranking algorithm. In the aspect of model architecture design, a hierarchical fusion framework is proposed: In the first level, multi-scale temporal feature extraction is realized by stacking dilation convolution, and feature fusion is carried out by combining hierarchical recurrent neural network; the second level constructs a joint time–frequency Transformer, divides low-frequency/high-frequency feature coding layers by convolution layers, establishes a cross-scale interaction mechanism to realize the mutual influence of frequency domain features, and deeply fuses the time coding results with frequency domain features. In the third level, a context-aware gating mechanism is introduced into a hierarchical gating loop unit (HGRNN), and dynamic gating signals are generated by attention weight calculation to realize adaptive screening and feature enhancement of key information. The validation of this method on the NASA C-MAPSS dataset shows that it has high RUL prediction accuracy.

To address key challenges in aeroengine RUL prediction—including nonlinear multi-scale degradation and dynamic inter-sensor correlations under time-varying load conditions—the main contributions of this study are reflected in three aspects, with distinct innovations compared to existing international research:

(1) An elastic sorting feature construction method based on time series indexes is proposed, breaking through the limitations of traditional fixed-weight feature engineering. Targeting variable-load operating conditions of aeroengines, this method realizes dynamic weight allocation and collaborative analysis of multi-channel sensor signals. Unlike existing methods that fail to adapt to dynamic degradation patterns, it effectively captures the evolving characteristics of time series data and avoids feature distortion caused by static fixed feature combinations, significantly enhancing the feature representation ability for complex dynamic degradation processes.
(2) A bidirectional cross-scale joint time–frequency transform interaction mechanism is designed, which realizes mutual modulation between low-frequency slow degradation trends and high-frequency transient fluctuation components via learnable 1D convolution gates. Distinct from existing time–frequency fusion methods that simply concatenate separate time-domain and frequency-domain feature maps, this dual-channel interactive architecture synchronously extracts long-term overall engine degradation drift and early weak fault oscillation information, overcoming the information loss defect of single-domain feature extraction.
(3) A context-aware hierarchical gated recurrent neural network architecture is constructed by embedding multi-head context attention gating signals into stacked recurrent layers. Unlike simple stacked shallow GRU/LSTM or pure self-attention Transformer modules, the hierarchical gating unit dynamically filters degradation-related key information during sequence forward propagation, enhances the model’s ability to capture long-range degradation dependencies under complex multi-operating conditions, and effectively alleviates the overfitting risk on datasets with multiple fault modes.

The ablation experiments in Section 3 fully verify the indispensability of the above three customized integrated mechanisms; removing any one module will lead to significant deterioration of RMSE and Score metrics on all four C-MAPSS subsets.

## 2. Related Studies

This chapter mainly describes the construction process of the whole prediction framework in detail, including multi-scale stack dilation convolution to extract features, time–frequency converter cross-scale interaction mechanism to enhance features, and hierarchical cyclic network context unit to capture long-term dependencies in the process of device degradation effectively.

### 2.1. Multi-Scale Stack Dilation Convolution

In engine degradation monitoring, time series data have many problems, such as multi-scale dynamics, long-distance dependence, non-stationarity and noise disturbance. Multi-scale stack dilation convolution can construct a super-wide receptive field covering multiple time steps by exponentially increasing the dilation rate combination to mine data features. The dilated convolution module only serves as a basic multi-scale feature extraction component, and its performance improvement relies on the subsequent elastic feature ranking preprocessing and cross-scale time–frequency interaction mechanism proposed in this paper. Let the expansion rate of the input data be 2, let the input data be *x*_0_. After passing through the convolution layer, the feature map *x*_0_ is obtained. The calculation process can be expressed as:

*x*_1_ = *ReLU*(*W*_1∗*d*=2_*x*_0_ + *b*_1_) (1)

where *W*_1_ is the weight matrix of the convolution kernel—it determines the linear combination of convolution operations on input features; *b*_1_ is the bias term, offset used to adjust convolution results; ∗*d* = 2 denotes convolution operation with expansion ratio 2; and ReLU as activation function, its role is to introduce nonlinear factors, so that the model can learn more complex feature patterns, avoiding the limitations of linear models. The subsequent convolution layer expansion rate increases correspondingly in turn.

The feature map set [X_1_,X_2_,X_3_,X_4_,X_5_] covers features at different scales. The feature map is obtained by the feature stitching operation X_concat_. Finally, a more concise and efficient fusion feature is obtained. The differences between different-scale features are eliminated, and more representative and robust feature information is extracted. Figure 1 is a multi-scale feature extraction diagram.

### 2.2. Cross-Scale Joint Time–Frequency Transform

Different from the conventional separated time–frequency decomposition operation in existing studies, the core innovation of this module is the designed bidirectional cross-frequency band feature interaction gate, rather than simple frequency domain splitting. In the signal-processing stage, a joint modeling method combining time-domain and frequency-domain features is designed: in the frequency-domain representation level, a multi-scale dependence extraction mechanism is constructed by using learnable adaptive frequency parameters, and key frequency components are screened by using parameter sparsity constraints; in the time-domain representation level, local features are dynamically extracted from adjacent data points with fixed length by using a sliding time window, to enhance the modeling ability of local relations of non-stationary signals [33]. Based on this, a cross-scale joint time–frequency transform model is proposed, depth coupling of two representations is realized through interactive calculation of the time–frequency coding matrix, and a cross-scale feature interaction mechanism is constructed. Figure 2 is a flow chart of cross-scale joint time–frequency change.

Aiming at the limitations of traditional time–frequency transform methods in cross-scale feature coupling, this study proposes a dual-channel coding architecture based on frequency components: The spectrum resolution is split into low frequency stationary components [34] with high-frequency transient components [35], independent coding is carried out through linear projection layers, and the dynamic modulation mechanism of the cross-frequency band is established [36].

Low frequency *L* is expressed as:

*L* = *Low*_*frequency encoding*(*X*) (2)

High frequency *H* is expressed as:

*H* = *High*_*frequency encoding*(*X*) (3)

It is necessary to clarify that the NASA C-MAPSS turbofan engine dataset adopted in this paper does not contain independent accelerometer vibration sensors, as summarized in Table 1. The term “high-frequency fluctuation component” used throughout this manuscript does not refer to directly measured vibration acceleration signals from dedicated vibration sensors. Instead, it represents virtual high-frequency periodic fluctuation components decomposed from multi-type physical sensor signals, including corrected fan speed NRf, corrected core speed NRc, LPC outlet total temperature T24 and HPC outlet static pressure P30.

The periodic oscillatory sub-signals separated from multi-physical sensor sequences are defined as high-frequency fluctuation components, which only reflect periodic numerical oscillation of sensor measurement data, without mapping to any specific mechanical fault of the engine. Based on the bidirectional time–frequency converter proposed in this work, we conduct sliding-window Fourier transform on normalized multi-sensor time series to separate two types of feature components:

(1) Low-frequency component L: Slow, steady drift trends of temperature and pressure parameters, which reflect the long-term overall performance degradation of the whole engine.
(2) High-frequency component H: Short-period periodic oscillatory sub-signals separated from the original sensor sequences.

Where *X* is the input signal. In order to fully explore the inherent correlation between high- and low-frequency features, a cross-scale feature interaction mechanism is introduced. Through a one-dimensional convolution layer, low-frequency features L are transformed to interact with high-frequency features *H*, and the effect of low-frequency features on high-frequency features can be expressed as:

*H*^′^ = *High*_*freq* + *Low*_*freq*(*L*,*H*) (4)

Similarly, the high-frequency feature *H* interacts with the low-frequency feature *L* via convolution, and the influence of the high-frequency feature on the low-frequency feature is:

*L*′ = *Low*_*freq* + *High*_*freq*(*H*,*L*) (5)

Meanwhile, considering the importance of signal-time characteristics, time series data are encoded to obtain time representation *T*:

*T* = *Time encoding*(*X*) (6)

The time representation and frequency features are fused, and after dimensional adjustment, the final fused features are obtained through linear layer combination processing with ReLU activation function:

*F* = *Final Fusion*(*L*′,*H*′,*T*) (7)

This method comprehensively analyses the time–frequency characteristics of signals through time–frequency coding, cross-scale interaction and time–frequency fusion.

### 2.3. Context-Based Gating Mechanism

This hierarchical recurrent structure is not a simple stack of standard GRUs; the core originality lies in the embedded context-aware dynamic attention gating mechanism specially designed for non-stationary engine degradation sequences. A context-aware mechanism is introduced into the basic architecture of the hierarchical cyclic gating unit: temporal hidden state and historical context vector are input into a multi-attention module in parallel, and dynamic context representation is generated by attention weight calculation. A gating signal-generation layer based on the context vector is added to the gating unit, and the Sigmoid function is used to adaptively adjust the input gate, forgetting gate and output gate. Finally, feature focusing is realized by an element-by-element product of the gating signal and the context representation. The hierarchical cyclic gating unit based on the context-gating mechanism is shown in Figure 3.

The module designs the HGRU architecture through the joint design of the attention mechanism and gate mechanism. It synchronously performs multi-channel linear mapping on the input feature matrix *x* and independently generates query vector *Q*, key vector *k*, and value vector *v*.

*Q* = *W_q_x* + *b_q_* (8)

where *W_q_* is the weight matrix of the query linear layer, and *b_q_* is the bias.

*k* = *W_k_x* + *b_k_* (9)

where *W_k_* is the weight matrix of the key linear layer, and *b_k_* is the bias.

*v* = *W_v_x* + *b_v_* (10)

where W_v_ is the weight matrix of the linear layer of value, and *b_v_* is the bias.

Reset gate:

*r_t_* = *σ*(*X_t_W_xr_* + *h_t_*_−1_*W_hr_* + *b_r_*) (11)

Update the door:

*Z_t_* = *σ*(*X_t_W_xz_* + *h_t_*_−1_*W_hz_* + *b_z_*) (12)

Candidate hidden state:

(13) $$\tilde{h_{t}}=\tanh(X_{t}W_{\mathrm{xh}}+r_{t}\cdot h_{t-1}W_{\mathrm{hh}}+b_{h})$$

Hidden status:

(14) $$h_{t}=\left(1-Z_{t}\right)\cdot \tilde{h_{t}}+Z_{t}\cdot h_{t-1}$$

Then, calculate the attention score:

(15) $${\mathrm{attn}}_{{\mathrm{scores}}_{i,j}}=\frac{q_{i}k_{j}^{T}}{\sqrt{d}}$$

where *d* is the dimension of the input feature and *i* and *j* denote positions in the sequence.

### 2.4. Prediction Framework

As shown in Figure 4, this model takes multi-sensor time series signals as inputs, processes them through cascaded feature-extraction modules, and finally outputs RUL prediction values and verifies performance based on evaluation indicators. The process includes: (1) Using the elastic sorting feature construction layer, the input signals are sorted dynamically by time series index, and the degradation-sensitive features are screened. (2) Using the multi-scale temporal sensing layer, a stack dilation convolution module is adopted to balance temporal resolution and receptive field range through exponential dilation rate configuration; (3) Time–frequency interaction enhancement layer: low-frequency/high-frequency coding channels are divided based on a joint time–frequency converter, and frequency domain feature interaction is realized by cross-scale convolution; (4) Context time series modeling layer: an attention weight gating mechanism is integrated through a hierarchical gating recurrent network to adjust the feature propagation path dynamically. Each module cascades to form an end-to-end prediction framework, and finally outputs RUL prediction results through the fully connected layer. Specifically, the elastic ranking algorithm of time series indicators acts as the front-end feature preprocessing module of the overall prediction framework, which adaptively reorders and weights multi-sensor time-domain, frequency-domain and statistical features before multi-scale stacked dilated convolution processing.

From the perspective of computational complexity, we analyze the resource consumption of each submodule of ERCG-HRNN. The elastic ranking feature construction module runs offline during data preprocessing, and all adaptive weighting and sorting calculations are completed in advance without additional computing overhead during online prediction. Stacked dilated convolution and cross-scale time–frequency fusion bring mild computational growth, which is essential for extracting multi-scale time–frequency coupled degradation features. Unlike Transformer-based models with heavy self-attention matrix multiplication, our recurrent-based architecture avoids sharp computation expansion with longer time series.

## 3. Experimental Study

This chapter constructs a prediction framework based on the C-MAPSS dataset, including elastic sorting of engine sensor features based on the time series index, multi-scale stack dilation convolution feature extraction module, multi-resolution time–frequency feature fusion module and hierarchical structure enhancement feature-extraction module. The model framework is verified by experiments using test sets, and the results are discussed and analyzed. Finally, the predictive performance of this model framework is compared with that of the newly developed model.

### 3.1. Dataset Description and Data Preprocessing

#### 3.1.1. Dataset

This paper presents an empirical analysis of the commercial modular aero-propulsion system simulation (C-MAPSS) dataset published by NASA [37]. The C-MAPSS dataset is highly simulative and accurately simulates the real degradation process of turbine engines. The data log for each engine is rich in data from 21 sensors, including engine number, operating cycle, and corresponding values for three system settings, altitude, throttle angle, and Mach number, which reflect engine-operating conditions [38].

The C-MAPSS dataset consists of several sub-datasets, each with its characteristics of operating conditions and fault types. Details of the dataset are shown in Table 1. FD001 presents one low-load condition with one turbine failure; FD002 contains six different load conditions from low load to extremely high load, and there is one compressor fault at the same time; FD003 contains a low-load condition and two different types of faults, fan failure and compressor failure; FD004 also contains the same six operating conditions as FD002, two fault types are compressor failure and turbine failure.

**Table 1:** Introduction to C-MAPSS datasets.

	FD001	FD002	FD003	FD004
Number of training set devices	100	260	100	249
Number of test set devices	100	259	100	248
Operating mode	1	6	1	6
Failure mode	1	1	2	2
Training set data volume	20,631	53,759	24,720	61,249
Test set data volume	13,096	33,991	16,596	41,214

The dataset is structurally divided into training, test, and validation sets. The training set records the whole degradation process data of the engine from initial operation to complete failure in detail, which provides rich learning samples for the model, so that it can fully understand the characteristics of each stage of engine degradation. The test set records the degradation data of the engine partial operation cycle to verify the model’s generalization ability in practical application. The validation set records each engine’s true remaining service life in the test set at the last operating cycle, which provides a key reference for evaluating the accuracy of the model prediction results.

Figure 5 shows a simplified schematic of an aircraft engine. The engine collected signals from 21 sensors in this study, as shown in Table 2. As the number of cycles increased, each engine failed, as evidenced by sensor data. In addition, the data collected by the sensor is contaminated by noise, and when the engine fails, the data collection will be interrupted.

In the early stage of the aeroengine’s operation, its performance degradation is not obvious, and it is still in a healthy operating state, so no degradation has occurred. Therefore, the segmentation label is usually adopted, and the RUL label remains unchanged during the initial operation phase when the engine runs stably. In the degeneration stage, the RUL value will decrease linearly until the time of failure, that is, the RUL value is 0, and the corrected RuL label formula is:

(16) $$R_{\mathrm{label}}=\left\{\begin{array}{c} R_{\mathrm{init}},R_{\mathrm{real}}>R_{\mathrm{init}}\\ R_{\mathrm{real}},R_{\mathrm{real}}<R_{\mathrm{init}} \end{array}\right.$$

*R_init_* represents RUL’s initial state and *R_real_* represents true RUL.

RMSE and Score are used to evaluate the predictive effect of the model. RMSE is the root mean square error. The smaller the RMSE value, the closer the RUL predicted by the model is to the true RUL value, and the higher the model’s efficiency. Score is an index proposed by the PHM08 data contest to evaluate the prediction performance of the RUL prediction model. The smaller the Score value, the higher the model efficiency. Its calculation formula is as follows:

(17) $$\mathrm{MSE}=\sqrt{\frac{1}{N}\sum_{i}^{N}{\left(\hat{y_{i}}-y_{i}\right)}^{2}}$$

(18) $$\mathrm{Score}=\sum_{i=1}^{N}\left\{\begin{array}{c} e^{-\frac{d_{i}}{13}}-1,d_{i}<0\\ e^{\frac{d_{i}}{10}}-1,d_{i}\geq 0 \end{array}\right.$$

Let *y_i_* denote the real RUL value and $\hat{y_{i}}$ denote the predicted RUL value. We define prediction error as $d_{i}=\hat{y_{i}}-y_{i}$. If *d_i_*<0, the model predicts earlier failure than the real situation; if *d_i_* ≥ 0, the model predicts later failure than the real situation.

#### 3.1.2. Data Preprocessing

Because of the different dimensions of each sensor parameter, it is difficult to obtain the expected result when analyzing the original sensor data, and the smaller measured value may be ignored, which leads to inaccurate analysis. In order to eliminate the influence caused by different sensor data units and improve the model’s calculation efficiency and accuracy, the sensor values are normalized by min-max normalization in this paper. Taking the FD001 dataset as an example, Figure 6 shows raw aircraft engine data, mapping the eigenvalues of the original data to the interval [0, 1], and Figure 7 shows the normalized data of the aircraft engine raw data. It can be seen from Figure 6 that some sensor signals do not change over time and cannot provide valid and valuable information for engine RUL prediction, so they are eliminated in subsequent models.

### 3.2. Feature Construction Based on Elastic Ranking of Temporal Metrics

All feature construction and elastic sorting operations in this section are performed on the 15 pre-screened valid sensors after removing constant-value invalid channels described in Section 3.1.2. For sensor data from each engine in the C-MAPSS dataset, we extracted horizontal and fluctuation indicators [39]. Calculating the mean value allows us to know the average level of each sensor data over some time, which helps grasp the general situation of each parameter under the normal operating state of the engine; standard deviation and variance are used to measure the dispersion of data. By analyzing these indicators, we can judge the stability of parameters in the engine-operation process. Standard deviation and variance are used to measure the dispersion of data. By analyzing these indicators, we can judge the stability of parameters in the engine-operation process [40], such as kurtosis and skewness. By comprehensively applying these horizontal indexes, fluctuation indexes and shape indexes to extract features from the C-MAPSS dataset, we can more comprehensively and deeply mine engine-running-state information in the data. Table 3 shows the effect of time series index feature construction.

In order to fully exploit the potential value of sensor data and improve the prediction accuracy of aeroengine RUL, a feature construction method for elastic ranking of time series indexes is proposed in the time series feature engineering of C-MAPSS datasets. This method can effectively reveal the multi-dimensional law of engine performance degradation under different operating conditions. The concrete implementation is descending-order arrangement, ascending-order arrangement, middle-value focus and low-value focus. Through structural reorganization of original data features, the input feature space with better identification and discrimination is constructed by analyzing the practical significance of physical parameters using traditional time series theory.

Table 4 compares the elastic ranking characteristics of time series indicators. All experiments adopt a fixed random seed to ensure reproducibility. After training for 250 epochs, we average the prediction metrics obtained from epoch 21 to epoch 250 as the final evaluation result to eliminate the fluctuation of early unstable training and the order of the original data. The RUL prediction results of each subset are quantitatively evaluated using the mean ± standard deviation. Data show that: FD002, FD003, and FD004 subsets use elastic sorting to construct features; their RUL prediction index is better than the original feature order; the FD001 subset of the original data order results is slightly better than elastic sorting for constructing features. Experimental results verify the validity of the proposed method for RUL prediction.

Based on the Pearson correlation coefficient calculation, we compare the correlation distribution between the original data features and the elastic sorted features. As shown in Figure 8, the left 1 is the original data feature, and the original feature association is relatively messy. After reordering (for example, the left 2 is focused and sorted according to the median value of time series index mean, and the right 1 is focused and sorted according to the median value of time series index standard deviation), the positive and negative correlations between engine residual life prediction and different features can be more clearly presented, which is convenient for rapid identification of key features. Image 2 on the left can identify the negative correlation between features 8 and 13 and life prediction, and the right 1 can highlight the negative correlation between features 10 and 13 and life prediction. Compared with the original features, the key information is more prominent.

Figure 9 shows the importance scores of 21 sensor features in the four subsets FD001-FD004 for engine residual life prediction. The figure shows that the sensor characteristics F11, F4, F13, F9, F14, F15, F21, and F6 have a great influence on engine life.

This study uses fixed-time window signals as input samples on the original data to effectively mine and apply the temporal correlation of data. The sample data constructed in this way contains time series information for both the current period and the historical period. The specific operation of the sliding window to generate sample data is shown in Figure 10.

Not all samples can satisfy the window division in the sliding-window setting. This paper calculates the mean and slope of linear fitting for each feature column to satisfy the window division for all samples. Manual features extracted from each sample are combined with the model-trained dataset to predict RUL.

Since window size is a key parameter of the time prediction task, it greatly affects the accuracy of RUL prediction. In this paper, the sliding window size is set to 10, 20, 30 and 40 to study the effect of window size on the model. To ensure fair comparison, the number of sensors is kept constant during the test to eliminate its effect on window size. Table 5 shows the effect of different window sizes on the FD001 and FD003 datasets, resulting in the model’s predictive performance for different window sizes.

Figure 11 shows the effect of different window sizes on the FD001 and FD003 datasets, resulting in the model’s predictive performance for different window sizes. When the window size is 30, the model achieves the best results. So 30 was chosen as the window size for this model.

Table 6 shows the effect of the step size of the 30 sliding window on the results. Taking FD001 and FD003 as examples, the results in the table show that the index mean result of the FD001 subset with a step size of 2 is good, but the dispersion degree of its standard deviation result is high, and there is instability. The mean result of the FD003 subset with a step size of 1 is the best. Finally, this experiment selected a step size of 1.

In order to make the model learn more abundant degenerate patterns and improve the generalization ability of the model, the training set samples are expanded, and the prediction model framework proposed in this paper and the data after elastic sorting of time series indicators are spliced with the original data to expand the samples. Table 7 shows the number of training samples after splicing.

In this experiment, four sorting methods, time series index mean and splicing with original data, are used to expand samples. Table 8 shows the index results of the four ranking methods.

It can be seen from Table 8 that when the median focus of the mean is spliced with the original data, both FD001 and FD002 subsets achieve good results, indicating that this feature construction method optimizes the local structure of the data and enhances the focus of the model on key features. Therefore, this method of splicing the median focus with the original data is finally selected in this paper.

The iteration number is a key parameter that has an important influence on the model’s performance. Too many iterations will increase training time and overfitting of the model network, and too few iterations will lead to underfitting of the model network. As shown in Figure 12, runs of 50, 100, 140, 170, 190, 220, 250, 260, 280 and 300 were used in this experiment. The model obtained the best results when the number of rounds was 250. So 250 was chosen as the final iteration number.

### 3.3. Feature Extraction from Multi-Scale Stack Dilation Convolution

This study constructs a five-layer one-dimensional expansive convolutional stacking network to mine data features from different scales. Let the input data be x_0_, then the feature mapping set is obtained by convolution-layer processing with expansion ratios of 2, 4, 8, 16 and 32 [X_1_,X_2_,X_3_,X_4_,X_5_], which covers features at different scales. Splicing the features obtains the feature map.

Convolutional layers are used for feature fusion. Therefore, redundant feature information is removed, and key feature information of different scales is effectively integrated. According to the comparison of multi-scale layer experimental results shown in Table 9, although the index results of the sixth layer convolution also show good performance results, with the increase in network layers, the model complexity increases exponentially, which not only significantly increases the consumption of computational resources and training time cost, but also may cause gradient disappearance or gradient explosion, resulting in complex convergence of the model, and the risk of overfitting is significantly increased. After comprehensive trade-offs of model performance, training efficiency and generalization ability, the five-layer convolutional architecture is finally selected.

### 3.4. Multi-Resolution Time–Frequency Feature Fusion

In this module, the input data is encoded in the frequency domain using a low-frequency bilinear layer and a high-frequency bilinear layer to generate low-frequency and high-frequency features, respectively. After input data enters the module, it is linearly transformed by two linear layers and mapped to different frequency spaces of low frequency and high frequency to obtain the frequency representation of low frequency and high frequency. In order to make the low-frequency features adapt to the convolution layer, the dimension is adjusted first. After further feature extraction and conversion by one-dimensional convolution, the original dimension is adjusted back and added with the high-frequency features to realize the influence of low frequency on high frequency; the same operation is done for the high-frequency features to make them influence the low-frequency features, to enrich the feature expression ability and help the model capture the complex relationship between different frequency scales. The latest time data is selected from the input data and mapped to the same dimensional space as the input channel through the time-coding layer to obtain the time representation. First, adjust the time representation dimension to match the frequency features, then add it to the low-frequency and high-frequency features to realize the initial fusion of time information and frequency features. The fused low-frequency and high-frequency features are spliced in the channel dimension to form feature vectors containing more information. Finally, the spliced feature vectors are input into a fusion layer composed of two linear layers and a ReLU activation function, and a nonlinear transformation obtains the final fusion features. Table 10 shows that when only low-frequency or high-frequency features are retained, the key indicators are significantly reduced; after introducing the two-way frequency interaction mechanism, the indicators perform optimally, verifying the effectiveness of complementary spatial features of different frequencies.

### 3.5. Hierarchical Structure Enhanced Feature Extraction

This study uses a hierarchical gated cyclic unit structure to process the multi-resolution fused features. During initialization, a three-layer network structure and corresponding context gating units are constructed according to feature dimensions, hidden layer dimensions and layers. In the forward propagation stage, input data passes through each layer sequentially. Each layer learns the time dependence relationship of the input sequence and outputs a feature representation, which then enters the context gating unit for processing. The obtained result is used as the input of the next layer to realize the fusion of feature information of different layers and time scales layer by layer. Finally, it outputs a representation synthesizing the features of each layer. As shown in Figure 13, the three-layer hierarchical gated recurrent unit achieves the minimal RMSE and Score for FD001 and FD003. For FD002 and FD004 with six complex variable operating conditions, shallow one/two-layer networks suffer severe underfitting due to insufficient capacity to fit multi-condition nonlinear degradation rules, while four-layer networks bring obvious overfitting risk and significantly increased parameter overhead and training time. Although individual metrics of the three-layer structure are slightly higher than extreme partial layers on a small number of test samples, the three-layer structure achieves the optimal comprehensive balance of prediction accuracy, generalization stability and computational complexity across all four datasets. We adopt a unified three-layer architecture for all subsets for two critical reasons:

(1) Cross-dataset robustness priority: The four C-MAPSS subsets cover single/multiple operating conditions and different fault modes. If we set different layer numbers for each dataset separately, the model loses generalizability and cannot be universally applied to unknown engine degradation datasets in practical PHM scenarios. A fixed unified network depth avoids dataset-specific hyperparameter tuning and enhances the practical engineering transferability of the proposed framework.
(2) Comprehensive performance trade-off based on dual metrics: We jointly evaluate RMSE and Score rather than relying on a single index. For FD002 and FD004, four-layer networks can marginally reduce RMSE but produce sharply increased Score values, which means the model generates large early prediction offsets in most test engines; one/two-layer networks have low computational cost but fail to capture long-range degradation dependencies, leading to overall poor prediction performance. The three-layer configuration maintains relatively low RMSE and Score simultaneously on all subsets, with the smallest standard deviation of prediction errors, reflecting stable and robust prediction performance.

In summary, the three-layer hierarchical gated recurrent unit is selected as the unified backbone structure for all subsequent experiments, which balances fitting capacity, anti-overfitting ability, cross-dataset generalization and computational overhead.

### 3.6. Experimental Verification and Performance Analysis

Figure 14 shows the predicted results for each subset of the C-MAPSS dataset, showing that the predicted RUL is close to the actual value. The prediction model constructed in this paper can effectively predict the RUL of aeroengines. The vertical axis represents RUL values, and the horizontal axis represents the number of engines tested. The blue curve represents the actual RUL, and the red curve represents the predicted RUL.

Figure 15 shows the distribution of prediction errors for data points recorded in the C-MAPSS dataset. For the FD001 subset, the errors are mainly distributed in the interval [−9, 10], for the FD002 subset, the errors are mainly distributed in the interval [−9, 10], for the FD003 subset, the errors are mainly distributed in the interval [−9, 10], and for the FD004 subset, the errors are mainly distributed in the interval [−19, 20].

To further observe and analyze the effectiveness of the prediction model in this paper, the degradation curves of different engines for each dataset were tracked separately. Figure 16 shows the results of degradation tracking curves for different engines in FD001-FD004.

It can be seen from the different engine-degradation-tracking curves in Figure 16 that since the increase in cycle period makes the engine fault characteristics more obvious and the degradation information richer, the model can fully learn these characteristics, enhance the fitting ability of RUL, and thus reduce the error. Therefore, in the early stage, the fluctuation difference between the predicted curve and the real curve is significant, and the model has limited accuracy in capturing the characteristics of the initial degradation stage; with the increase in the period, the predicted curve gradually approaches the real curve, the fluctuation decreases, and the prediction error decreases significantly. The models using the FD001 and FD003 datasets exhibit higher prediction accuracy than those using the FD002 and FD004 datasets. The above study suggests this difference may be because sub-datasets FD001 and FD003 were acquired only under a single operating condition. In contrast, sub-datasets FD002 and FD004 were acquired under up to six operating conditions. The more complex the operating conditions are, the more complex the corresponding degradation process will be, so it will significantly increase the difficulty of accurately predicting RUL.

Table 11 shows how the model in this paper compares with DCNN&BiGRU [41], IMDSSN [42], SDFEPN [30], RMSE, and Score metrics for multiple existing methods on FD001-FD004 datasets. Given that the time series dataset exhibits stationary characteristics and relatively stable fluctuations, the final prediction results in this paper adopt the format of mean plus or minus standard deviation to reflect the core trend and reasonable fluctuation range of the data. All models are trained with a fixed random seed for reproducibility. Each training process runs for 250 epochs, and we average the evaluation metrics from epoch 21 to epoch 250 to mitigate the unstable performance caused by early-stage training fluctuations. By numerical comparison, we can see that the performance of this model is optimal, exceeding most of the existing methods. Specifically, the proposed method achieves the lowest RMSE across all datasets. Demonstrating that the reduction in RMSE is statistically significant and not attributable to random experimental variations. Moreover, the proposed method exhibits smaller standard deviations in RMSE compared to comparative methods, indicating more stable performance under different experimental conditions.

In this work, we mainly focus on lightweight prognostics frameworks applicable to on-board aeroengine monitoring equipment with limited computing resources, so the comparative baselines in Table 11 are selected from widely deployed CNN, RNN and hybrid neural network methods in industrial predictive maintenance. Recently, Transformer and foundation model-based RUL prediction approaches have drawn wide attention in PHM research, yet such architectures usually introduce massive parameter overhead and high inference latency, which restricts their real-time deployment on edge airborne devices. Different from vanilla Transformer encoders that rely solely on self-attention to capture long-range dependence, our ERCG-HRNN integrates hierarchical recurrent gating units and cross-scale time–frequency interaction modules, which achieves competitive prediction accuracy with much lower computational cost under multi-variable non-stationary operating conditions. Although Transformer-based methods are not included in the quantitative comparison table due to the lightweight positioning of this study, the inherent differences, application advantages and deployment limitations between the proposed method and existing Transformer prognostic models are analyzed qualitatively above to clarify the research positioning of our framework.

This paper tests the contribution of the time–frequency converter cross-scale interaction module and hierarchical cyclic network context module to RUL prediction. It can be seen from Table 12 that the fourth to sixth columns correspond to the cases of no feature structure, no time–frequency interaction and no hierarchical cyclic context module, respectively. The results show that the test performance decreases when any module is missing. Although some RUL indexes of the FD004 subset seem excellent when there is no frequency interaction module, the standard deviation is large, and the stability is poor. The three factors contribute significantly to RUL prediction and provide multi-dimensional support for model performance improvement.

## 4. Conclusions

This paper proposes a novel framework for the accurate prediction of aeroengine RUL, a critical task in aircraft PHM.

The framework constructs an elastic sorting feature system for multi-channel sensor signals based on time series indexes, and achieves comprehensive extraction of multi-dimensional degradation-sensitive features through the proposed elastic ranking algorithm of time series indicators. This innovative elastic sorting method effectively captures the dynamic evolutionary characteristics of time series data under time-varying variable operating conditions across distinct time steps—a key limitation of conventional fixed-weight feature engineering approaches. By resolving this challenge, the method significantly enhances the prediction model’s capability to fuse multi-source heterogeneous sensor data, which is essential for handling the diverse data types generated by on-board aeroengine monitoring systems.

At the feature enhancement level, the framework introduces a joint time–frequency transform and cross-scale interaction mechanism. This module realizes multi-dimensional feature decoupling of complex degraded signals via joint time–frequency domain analysis and cross-scale feature interaction, addressing the inadequacy of single-domain feature extraction methods in capturing complementary time–frequency information. Specifically, it enables accurate extraction of multi-scale dynamic features during the nonlinear degradation process of aeroengines, thereby providing high-resolution time–frequency domain input for the prediction model and further improving the accuracy and reliability of RUL prediction.

In terms of model construction, the framework designs contextual units for hierarchical recurrent networks. Through a multi-level recurrent structure, this module achieves deep contextual modeling of time series data, effectively capturing long-term dependencies in equipment degradation while preserving temporal continuity. This hierarchical design not only enhances the model’s ability to recognize complex degradation patterns but also strengthens the robustness and generalization performance of prediction results.

Experimental results demonstrate that the prediction error of the proposed framework is significantly reduced under non-stationary degradation scenarios—a common challenge in practical aeroengine operation. This outcome verifies the framework’s practical application value in the field of aeroengine health management, laying a foundation for its potential integration into industrial PHM systems.

In future work, we will conduct lightweight optimization for the proposed framework, including sliding-window size adjustment, network-layer pruning and low-rank feature approximation. We will further quantitatively analyze model parameters, training overhead and inference latency, and complete comparative efficiency experiments with mainstream prognostic algorithms to explore edge-device deployment schemes.

Moreover, we will extend our experimental datasets beyond C-MAPSS, including rolling bearing degradation datasets and different turbofan simulation datasets, to comprehensively validate the generalization capacity of our method on diverse mechanical degradation tasks.

## Figures and Tables

**Figure 1:**
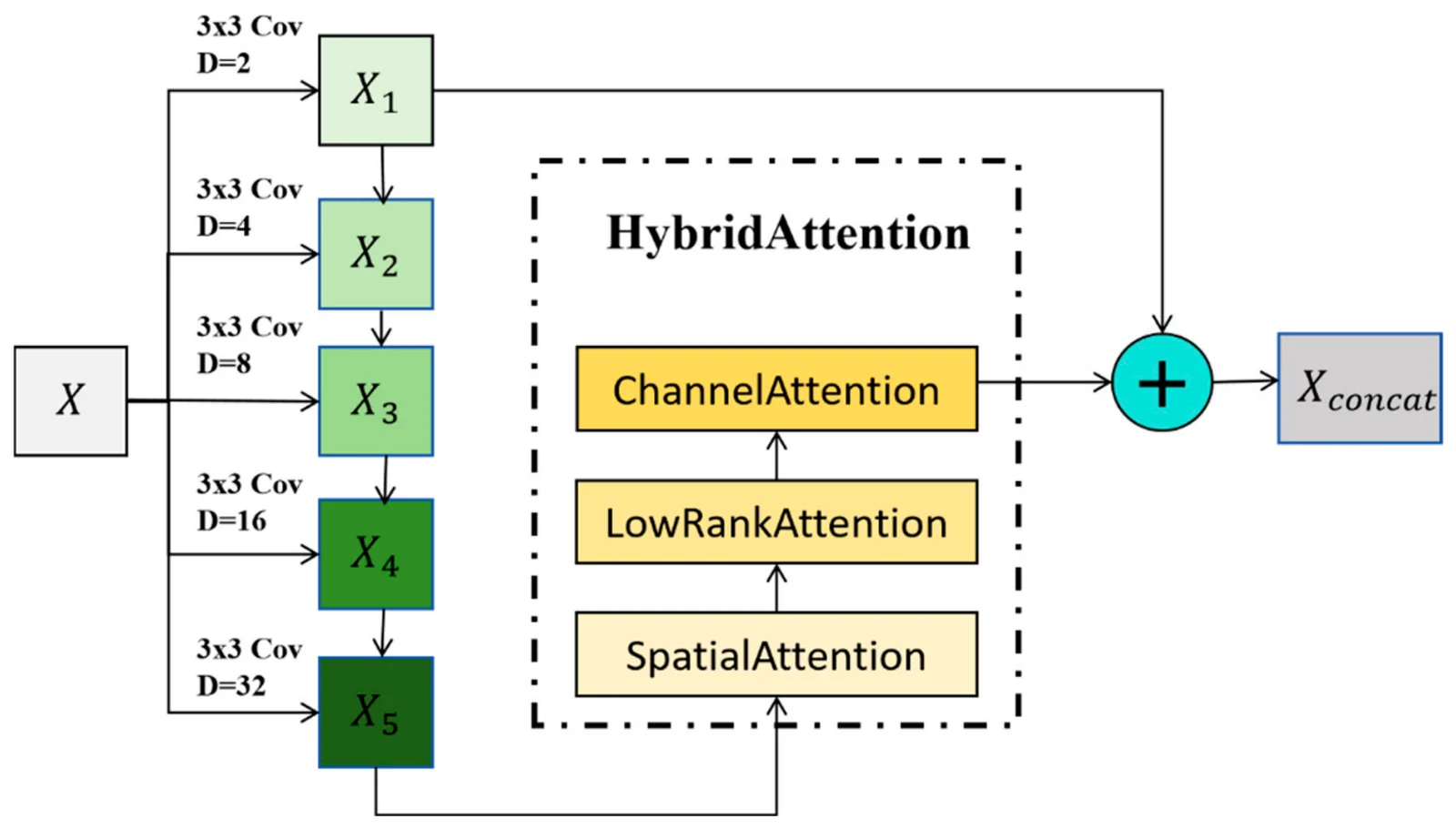
Multi-scale feature extraction diagram.

**Figure 2:**
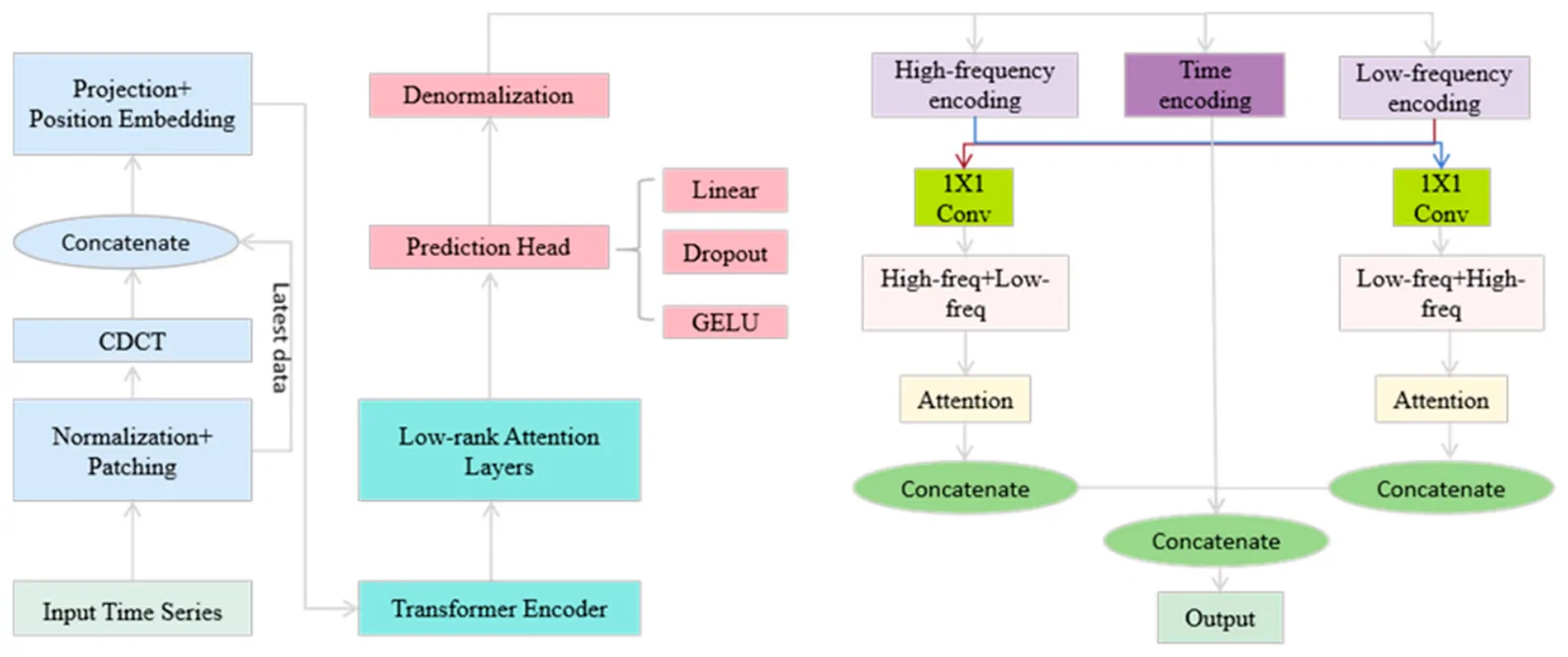
Flow chart of cross-scale joint time–frequency variation.

**Figure 3:**
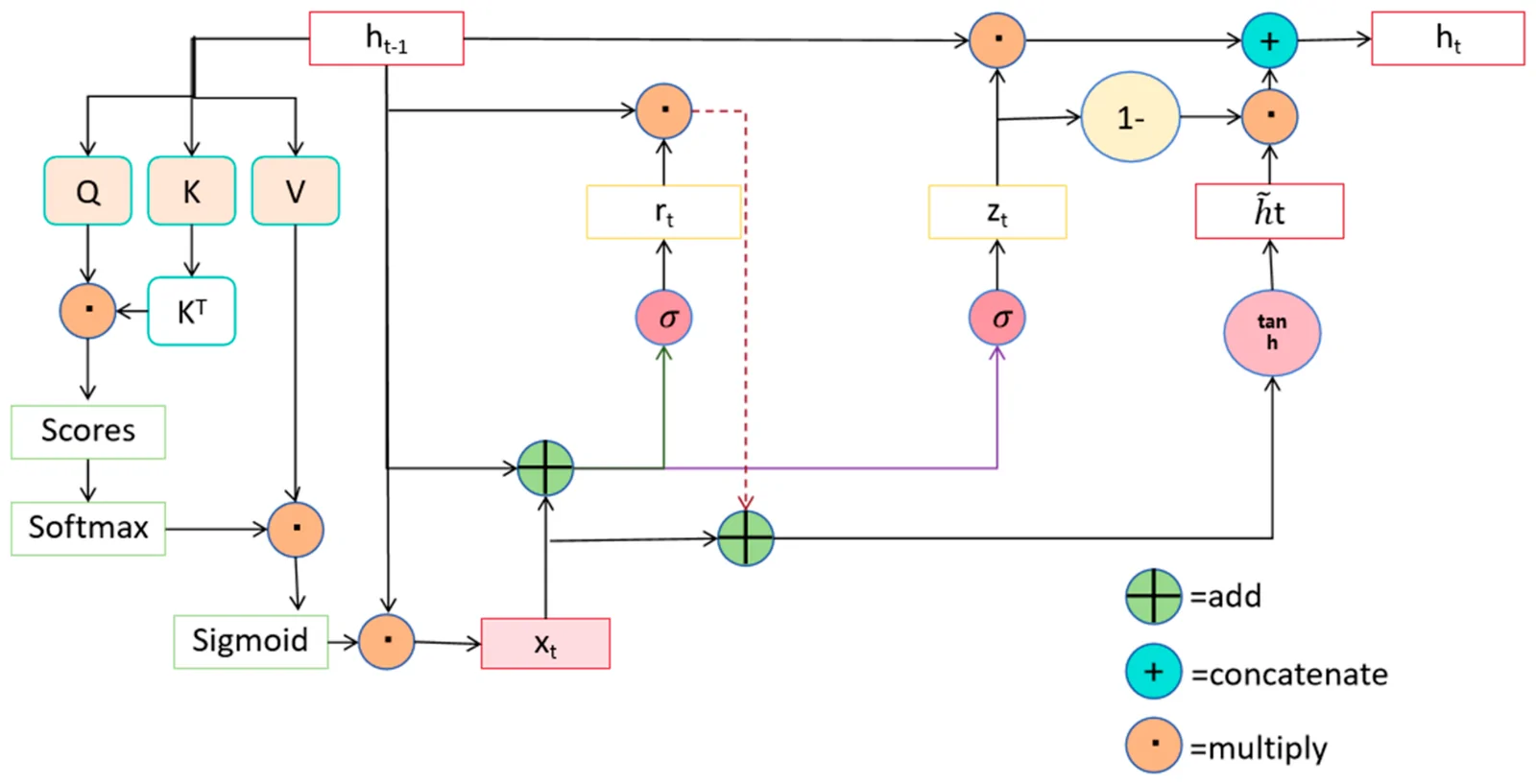
Hierarchical cyclic gating unit based on context-gating mechanism.

**Figure 4:**
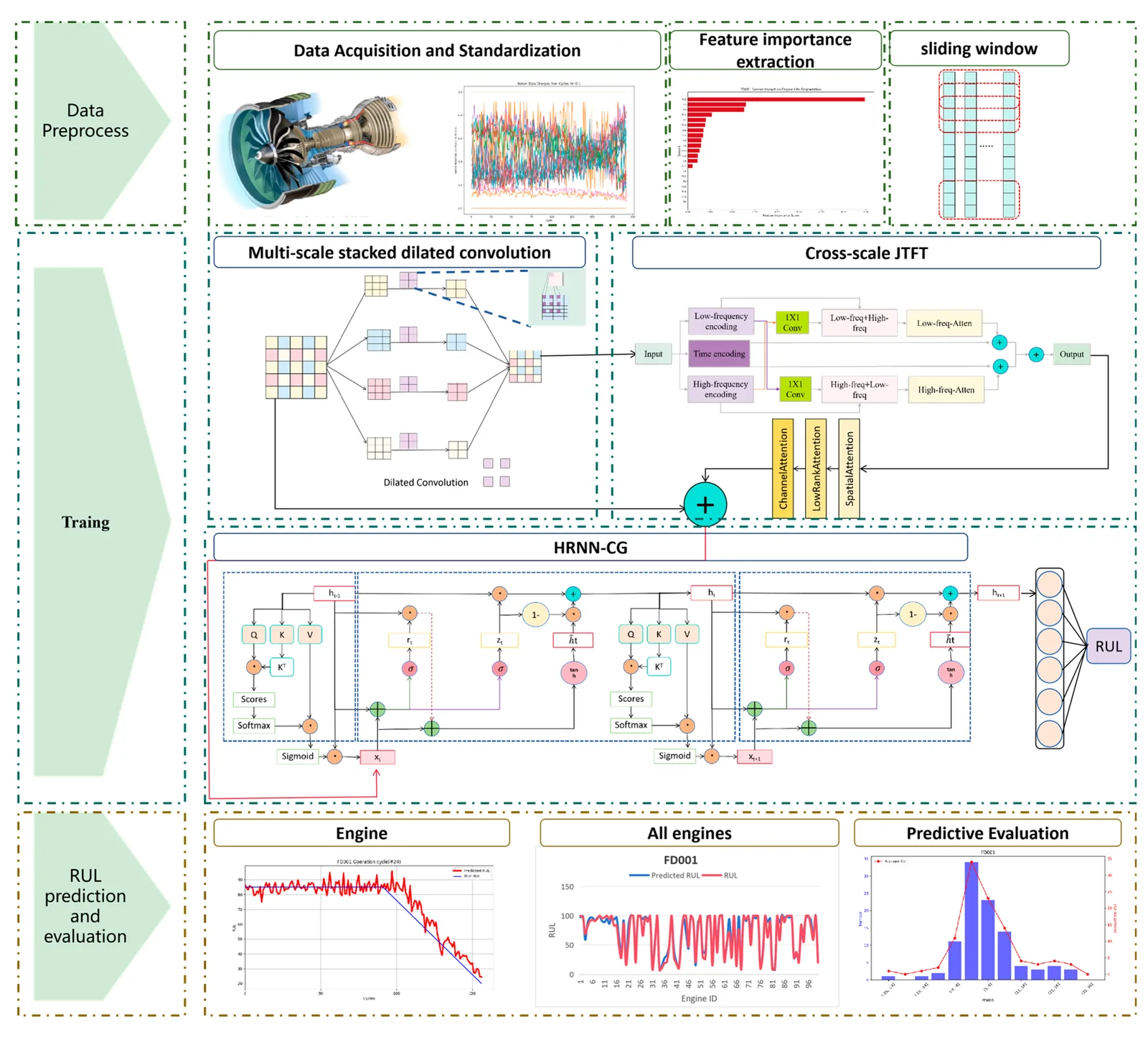
Overall prediction model framework.

**Figure 5:**
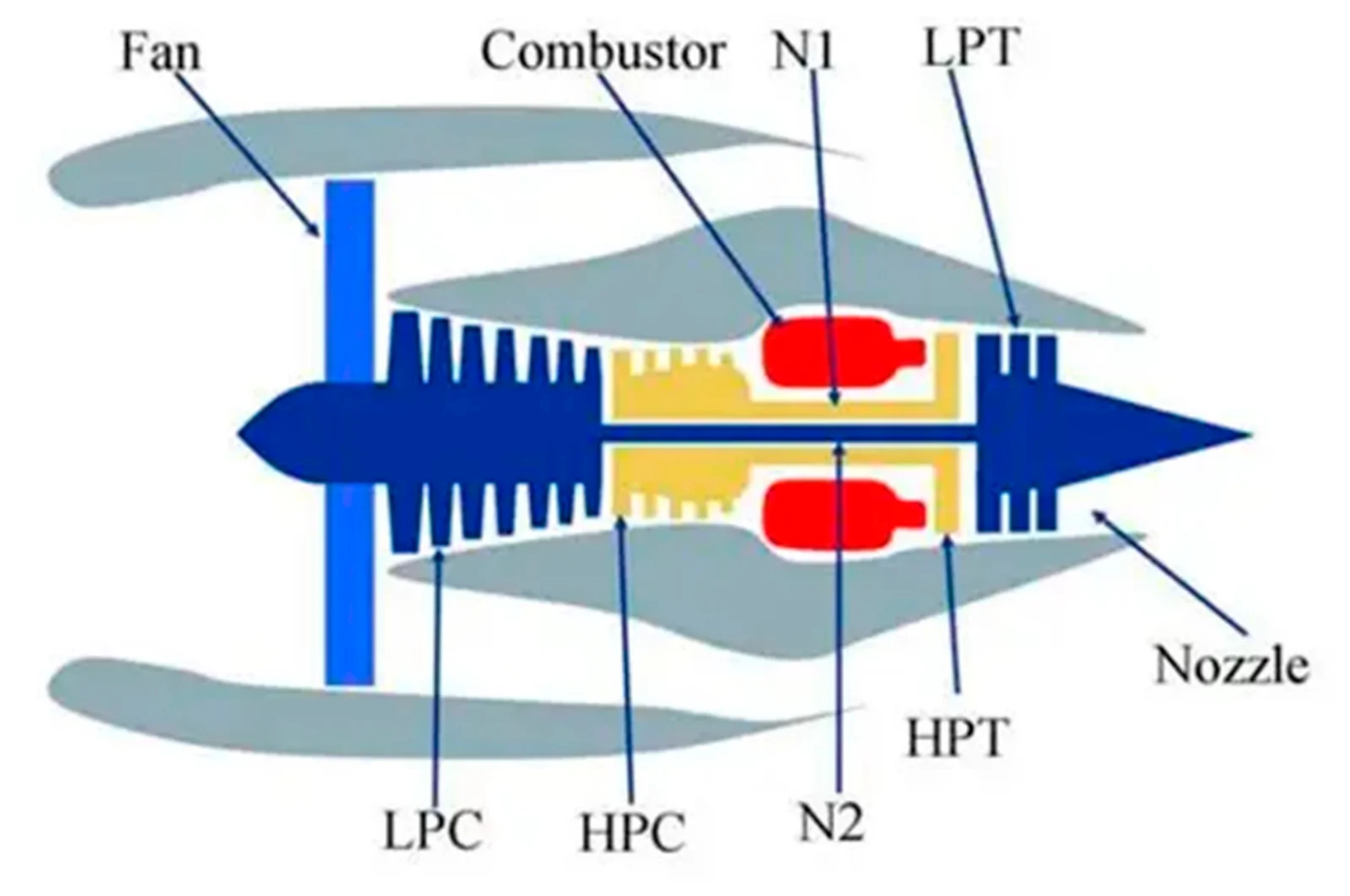
Simplified schematic diagram of the engine.

**Figure 6:**
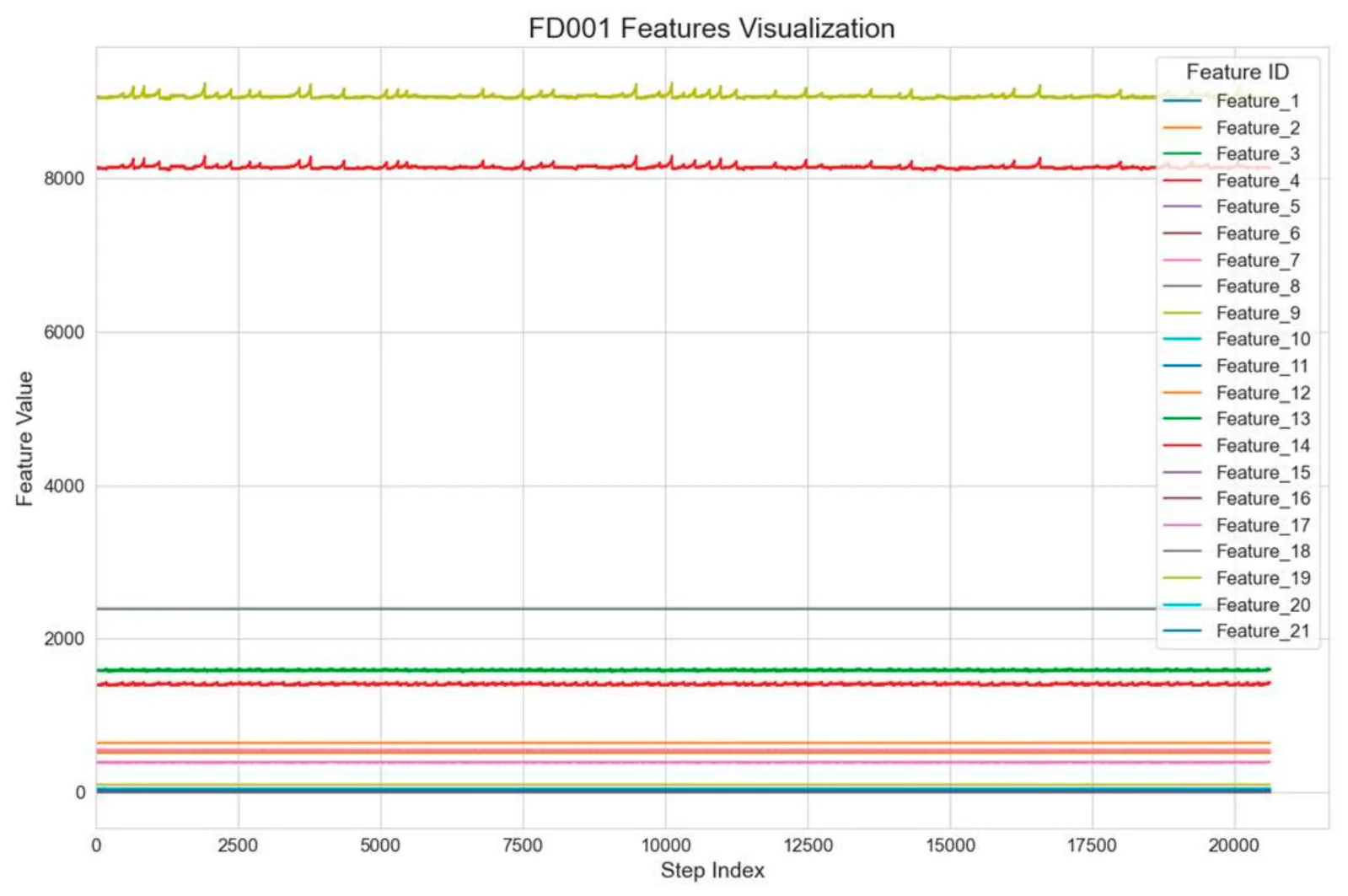
Raw data signal.

**Figure 7:**
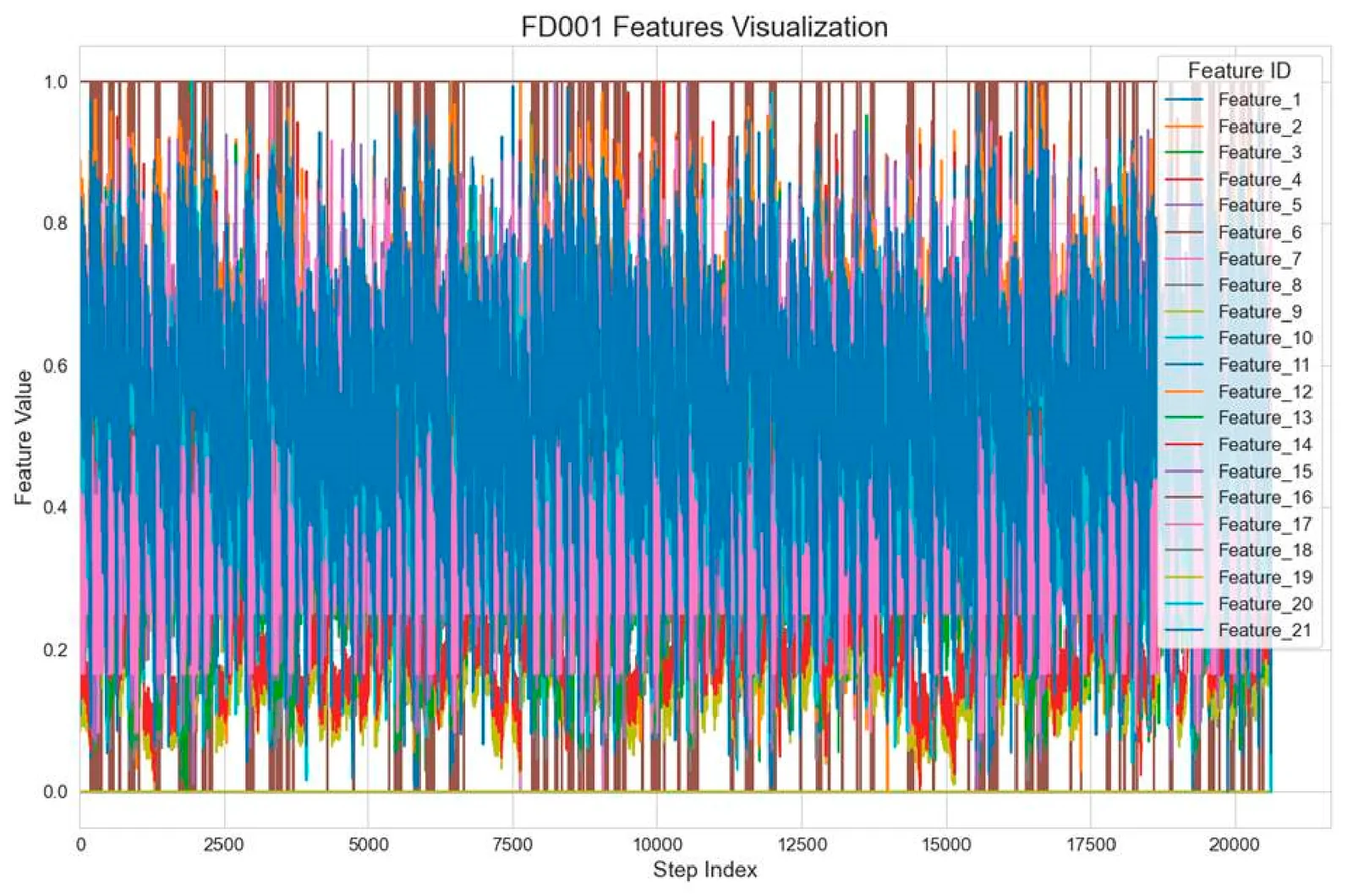
Normalized data.

**Figure 8:**
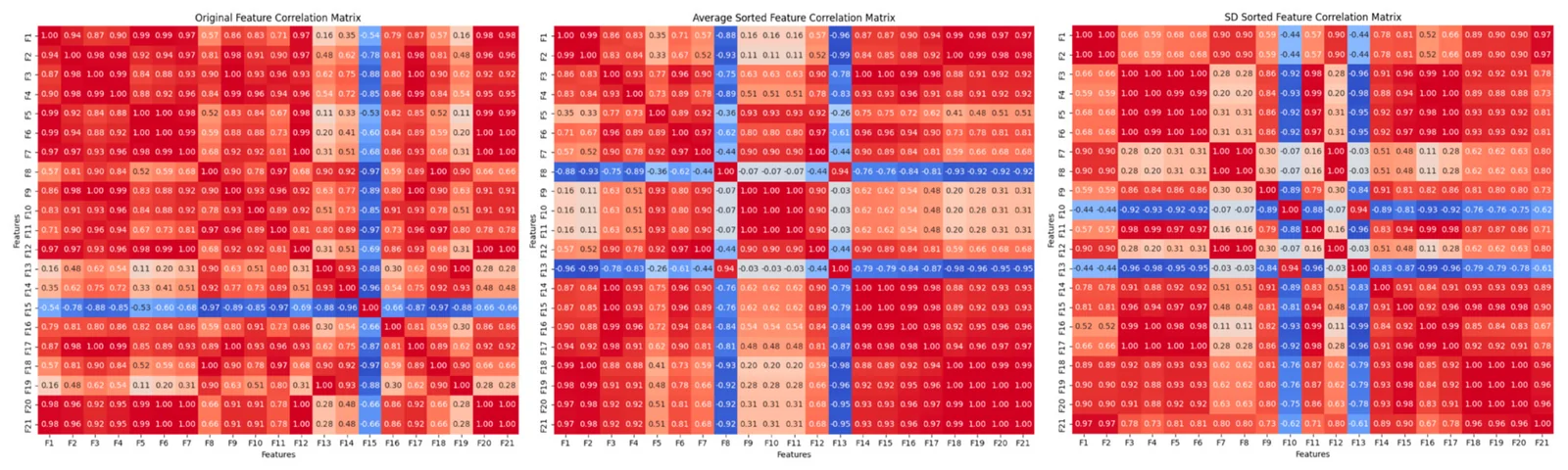
Comparison between original data and characteristic structure features.

**Figure 9:**
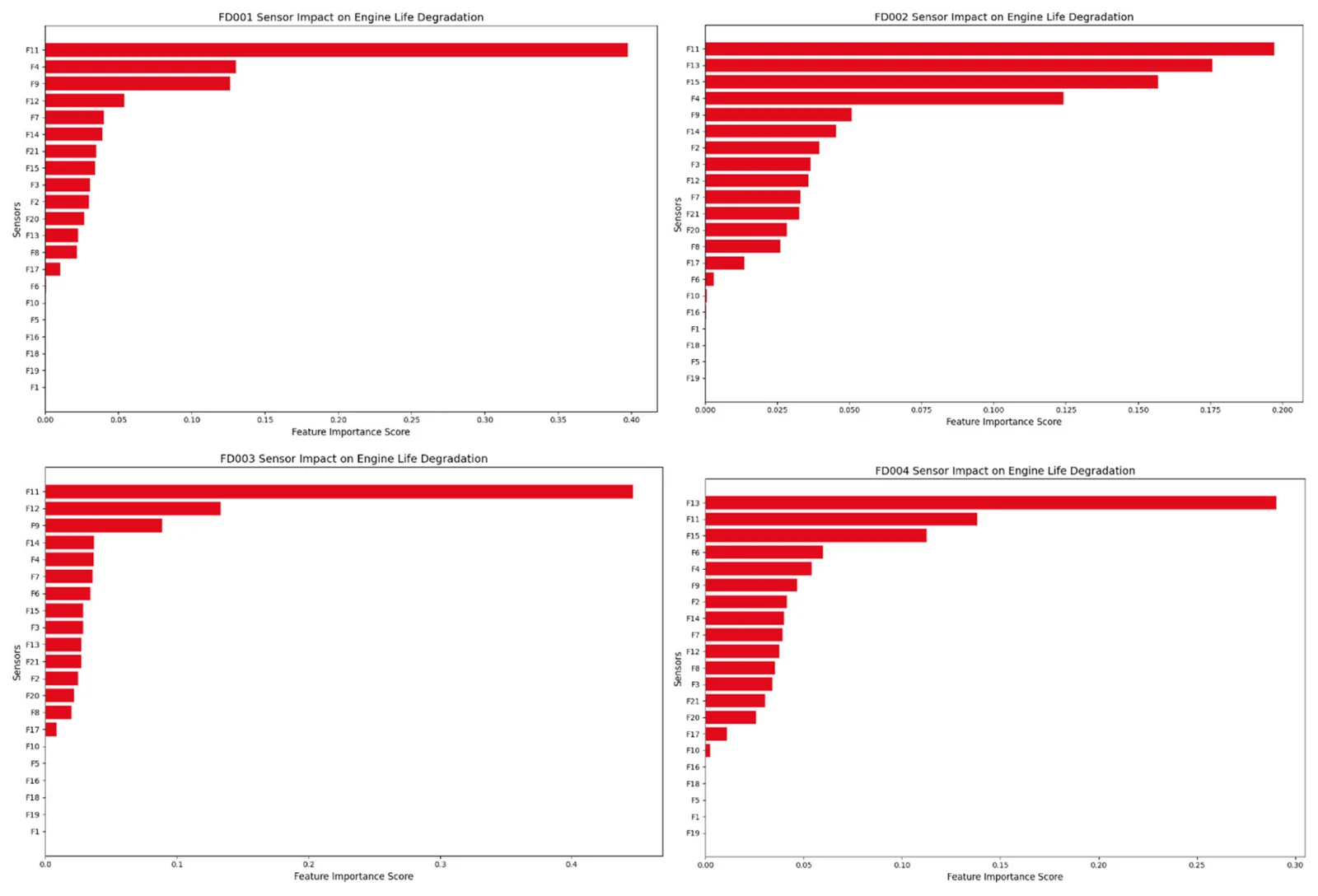
Feature importance score.

**Figure 10:**
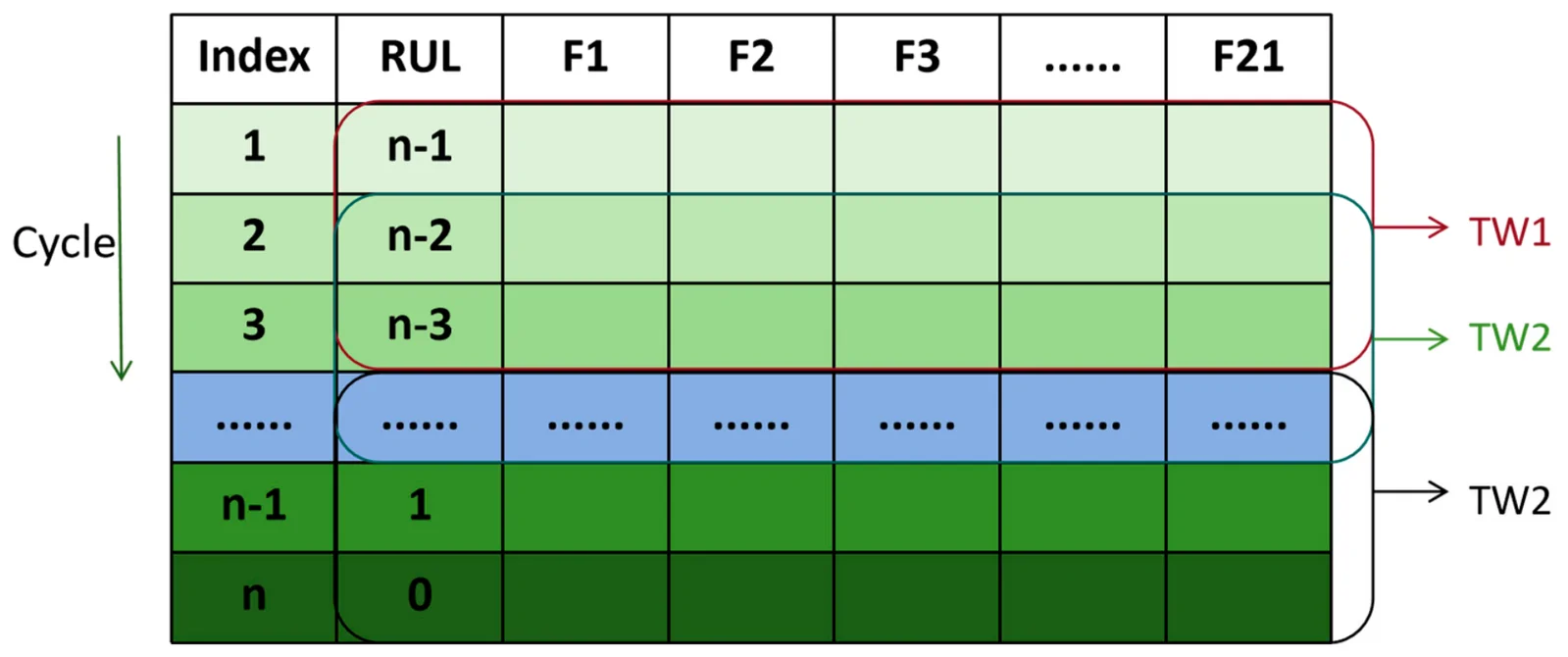
Schematic diagram of sliding window.

**Figure 11:**
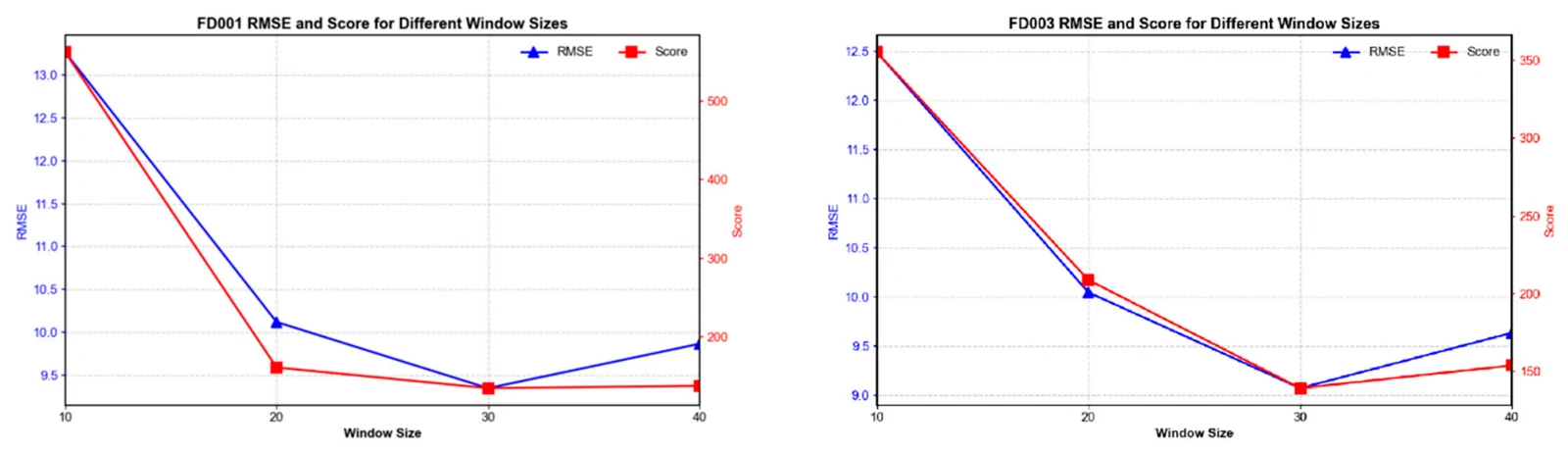
Comparison of results with different window sizes.

**Figure 12:**
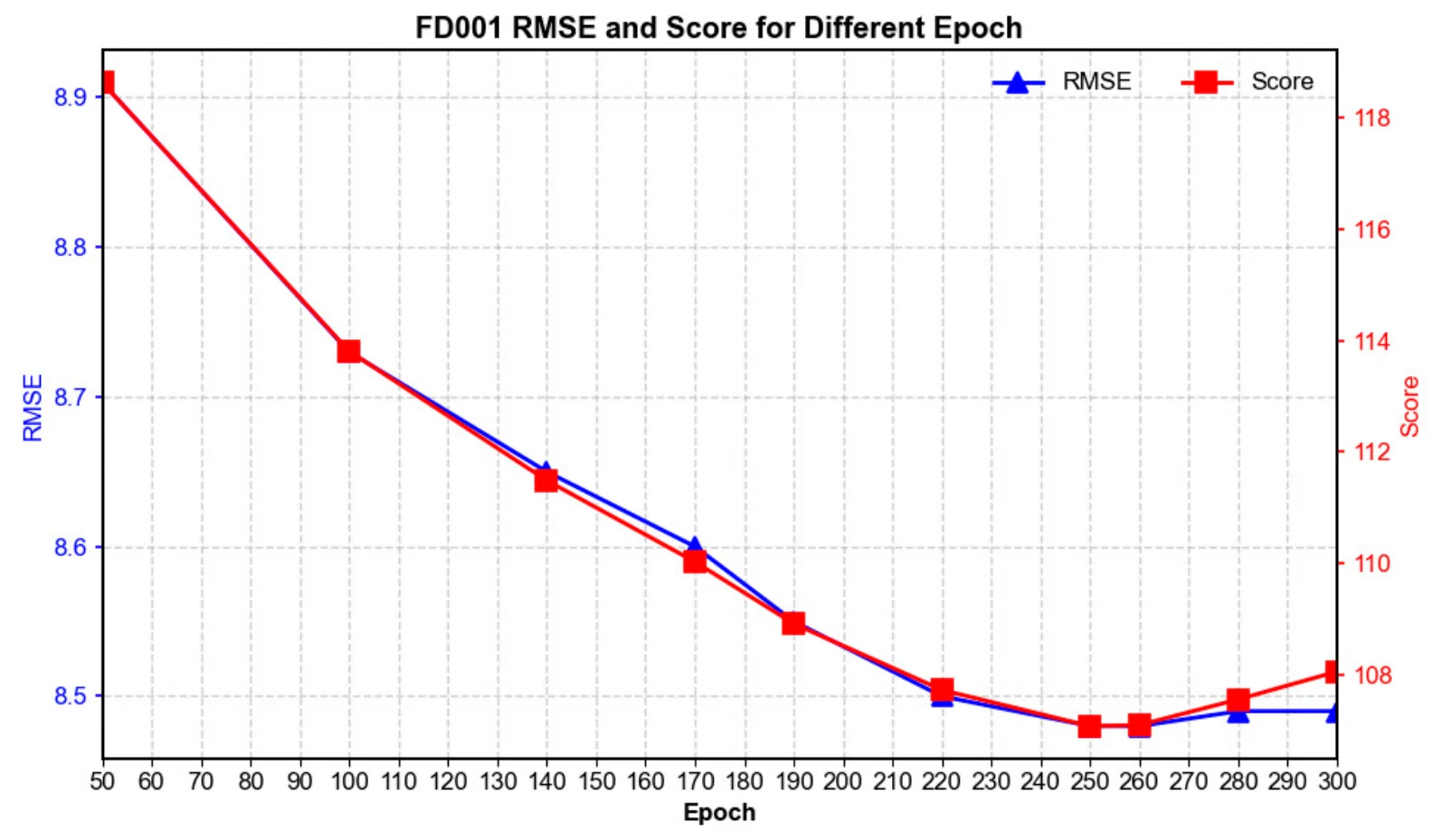
Comparison of different epoch results.

**Figure 13:**
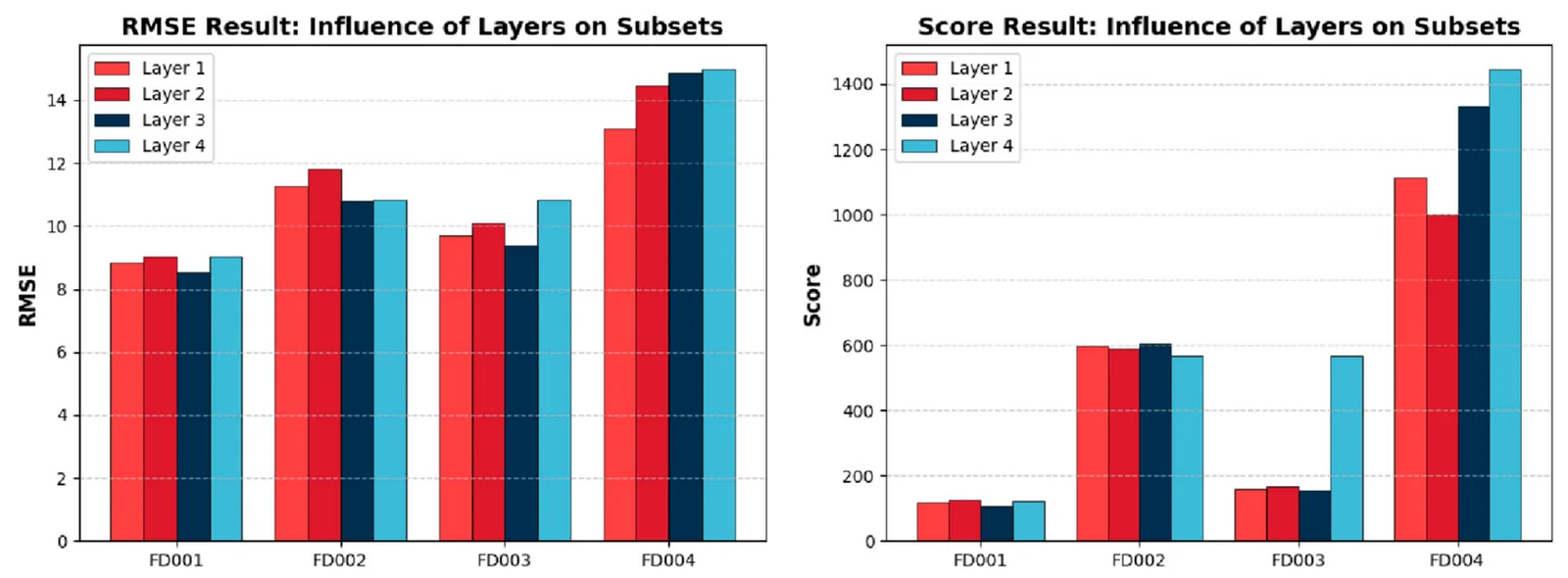
Comparison of experimental results of the GRU layer.

**Figure 14:**
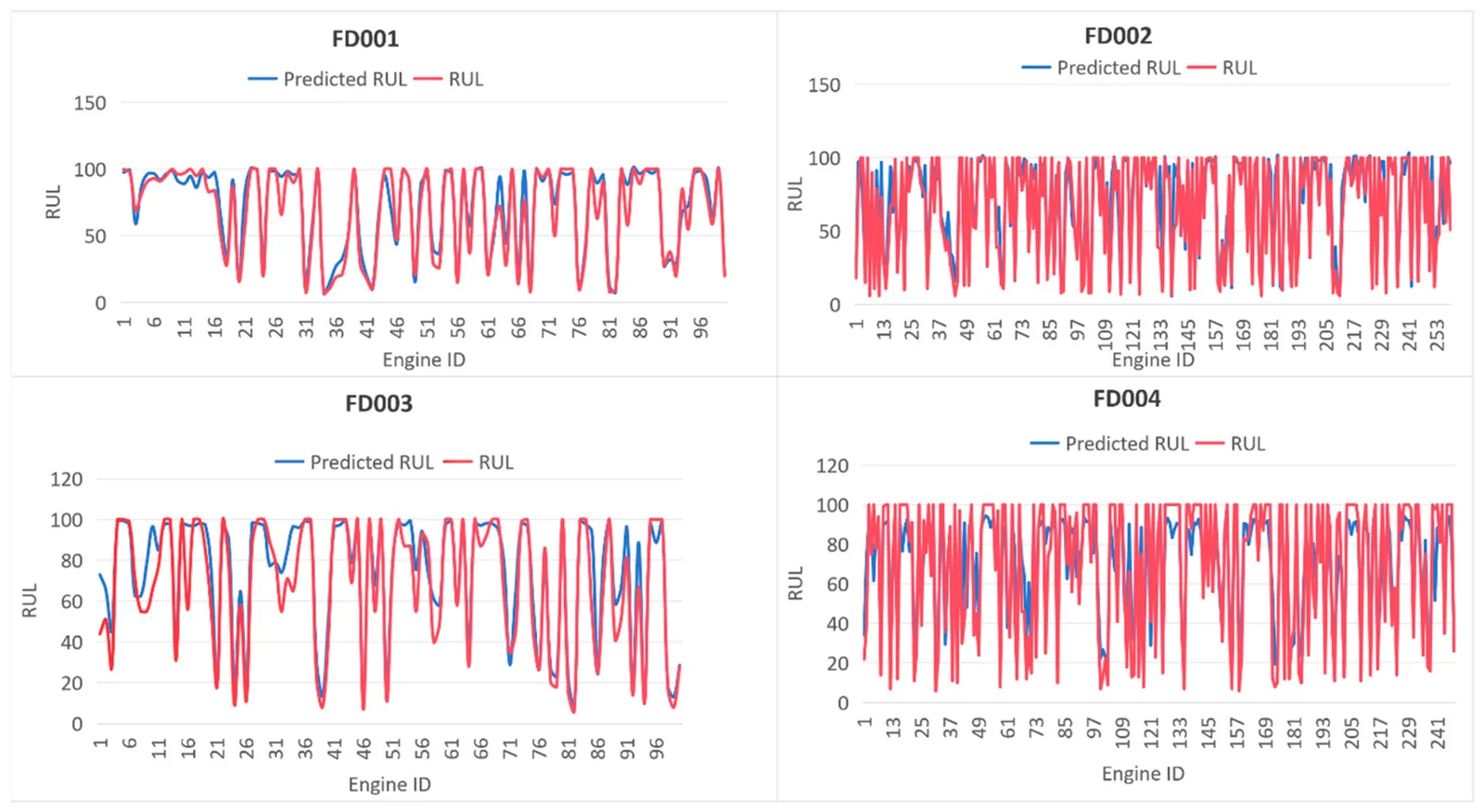
FD001-FD004 RUL actual vs. predicted.

**Figure 15:**
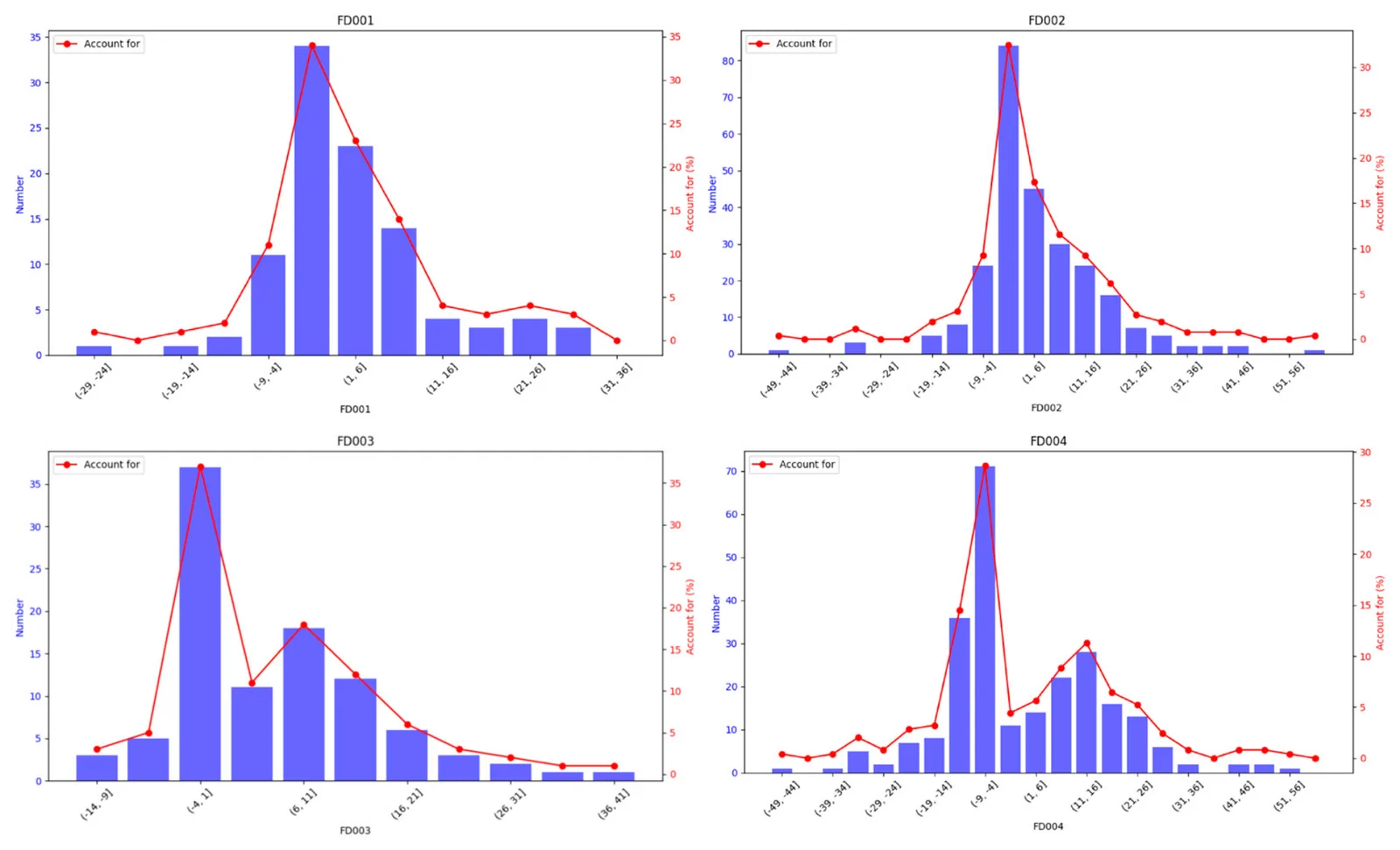
Error distribution FD001-FD004 for RUL prediction of test engine.

**Figure 16:**
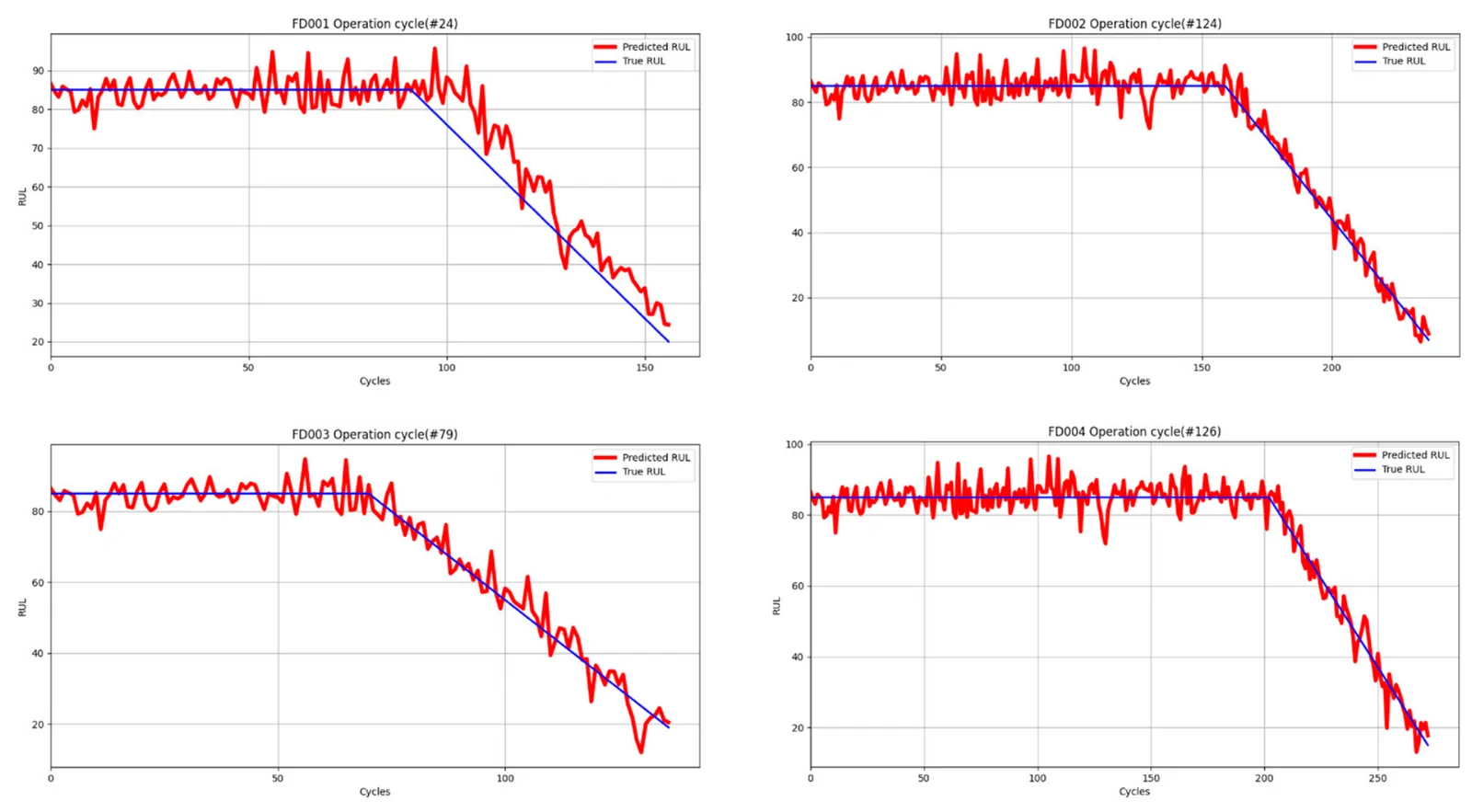
RUL prediction results FD001-FD004 for four examples of test engines.

**Table 2:** Sensor information.

Sensor	Symbol	Description	Sensors	Symbol	Description
1	T2	The total temperature at the fan inlet	12	Phi	Ratio of fuel flow to Ps30
2	T24	The total temperature at the LPC outlet	13	NRf	Corrected fan speed
3	T30	The total temperature at the HPC outlet	14	NRc	Corrected core speed
4	T50	The total temperature at the LPT outlet	15	BPR	Bypass ratio
5	P2	Pressure at the fan inlet	16	farB	Burner fuel–air ratio
6	P15	Total pressure in the bypass duct	17	bleed	Bleed enthalpy
7	P30	Total pressure at the HPC outlet	18	Nf_dmd	Demanded fan speed
8	Nf	Physical fan speed	19	PCNfR_dmd	Demanded corrected fan speed
9	Nc	Physical core speed	20	W31	HPT coolant bleed
10	per	Engine pressure ratio (P50/P2)	21	W32	LPT coolant bleed
11	Ps30	Static pressure at the HPC outlet			

**Table 3:** Effect of time series indicators.

Timing Indicator	Indicator Type	Contribution to the Analysis of Engine-Operating Conditions
Mean value	Level indicator	Understand the average level of each sensor dataset over a period of time and grasp the general situation of each parameter under normal engine-operating conditions.
Standard deviation	Volatility indicator	Measure the data dispersion and judge the parameters’ stability during engine operation. The larger the standard deviation, the more scattered the data and the worse the stability may be.
Variance	Volatility indicator	Measuring the degree of dispersion of the data is similar to the effect of standard deviation. The greater the variance, the greater the degree of dispersion of the data, which can assist in judging the stability of engine parameters.
Kurtosis	Morphological indexes	Understanding the symmetry of the data distribution can help determine the direction and characteristics of parameter fluctuations during engine operation. A skewness value other than zero means that the data distribution is asymmetrical.

**Table 4:** Comparison of original data sequence and feature construction results.

	FD001		FD002		FD003		FD004	
	RMSE	Score	RMSE	Score	RMSE	Score	RMSE	Score
original order	9.3501 ± 1.16382	134.2417 ± 27.8335	12.8997 ± 0.45	812.5818 ± 74.306	9.0752 ± 1.0320	139.1394 ± 27.7291	16.3716 ± 0.6048	1844.0264 ± 306.4307
descending order of standard deviation	9.4722 ± 1.0319	138.7315 ± 25.3196	10.5874 ± 1.3989	550.9756 ± 230.4330	8.6875 ± 1.1480	126.9041 ± 33.4928	12.4517 ± 1.7128	899.6254 ± 792.5117
ascending order of standard deviation	9.7463 ± 1.2559	143.8166 ± 28.0838	10.6716 ± 1.4647	543.6209 ± 194.4025	9.0884 ± 0.9934	135.5482 ± 28.0991	12.3559 ± 1.4643	850.0442 ± 725.6472
standard deviation, median focus	9.5640 ± 1.1191	140.1365 ± 25.1153	10.8409 ± 1.5338	576.3122 ± 239.8997	8.8708 ± 1.2739	130.8953 ± 37.7126	12.0561 ± 1.5579	832.7256 ± 782.2360
low set of standard deviations	9.7215 ± 0.8801	145.7540 ± 23.1833	10.7788 ± 1.6585	551.9272 ± 219.0405	8.7624 ± 1.1867	127.1814 ± 33.3282	12.0189 ± 1.9039	814.5341 ± 759.5130
descending order of mean	9.6073 ± 1.2053	141.7545 ± 29.1144	10.6003 ± 1.3997	571.0582 ± 220.1679	8.8198 ± 1.0740	130.8226 ± 31.4079	12.2854 ± 1.5305	903.7478 ± 781.0785
ascending order of means	9.7089 ± 1.1709	142.6397 ± 24.9387	10.8762 ± 1.4601	570.2713 ± 229.0574	9.0456 ± 1.0019	134.5592 ± 29.6778	12.3415 ± 1.7976	833.0439 ± 723.7454
mean median focus	9.6296 ± 1.0110	142.2 ± 24.0749	11.0008 ± 1.7284	560.7017 ± 221.0929	8.8556 ± 1.2572	129.4435 ± 34.0962	12.3346 ± 1.7667	909.0653 ± 891.5194
concentration of mean low values	9.6940 ± 1.1928	143.9630 ± 27.1364	10.8673 ± 1.5081	581.1723 ± 244.9914	8.9776 ± 0.9841	134.7639 ± 28.6689	12.2286 ± 1.6344	870.7588 ± 1099.2388

**Table 5:** Sliding window parameter setting results.

Subsets	Time Window Size	Step Size	Training Samples	Test Samples	RMSE	Score
FD001	10	1	19,731	100	13.2664 ± 0.4289	562.8556 ± 126.1138
FD001	20	1	18,731	100	10.1213 ± 0.8553	160.6991 ± 42.4233
FD001	30	1	17,731	100	9.3501 ± 1.16382	134.2417 ± 27.8335
FD001	40	1	16,731	100	9.8674 ± 0.7417	137.2618 ± 18.9671
FD003	10	1	23,820	100	12.4919 ± 0.5580	355.4252 ± 54.8581
FD003	20	1	22,820	100	10.0489 ± 1.5193	208.7683 ± 69.5126
FD003	30	1	21,820	100	9.0752 ± 1.0320	139.1394 ± 27.7291
FD003	40	1	20,820	100	9.637 ± 0.3093	153.7511 ± 14.9462

**Table 6:** Sliding-window step parameter setting results.

Subsets	Time Window Size	Step Size	Training Samples	Test Samples	RMSE	Score
FD001	30	1	17,731	100	9.3501 ± 1.16382	134.2417 ± 27.8335
FD001	30	2	8890	100	9.1496 ± 2.4923	127.0189 ± 59.0619
FD001	30	3	5947	100	10.1787 ± 2.9031	149.3706 ± 69.9519
FD003	30	1	21,820	100	9.0752 ± 1.0320	139.1394 ± 27.7291
FD003	30	2	10,933	100	10.0802 ± 1.2457	171.4116 ± 44.9905
FD003	30	3	7309	100	10.7184 ± 1.1677	189.3666 ± 41.2436

**Table 7:** Number of training samples for data enhancement.

Subsets	Time Window Size	Step Size	Training Samples	Test Samples
FD001	30	1	35,462	100
FD002	30	1	92,438	259
FD003	30	1	43,640	100
FD004	30	1	108,056	248

**Table 8:** Comparison of results of different feature structures.

	FD001		FD002		FD003		FD004	
	RMSE	Score	RMSE	Score	RMSE	Score	RMSE	Score
Descending permutation	9.2008 ± 0.8029	135.8837 ± 35.8399	12.6139 ± 1.0254	829.0646 ± 251.8142	9.6333 ± 1.3543	163.9454 ± 68.3207	15.3315 ± 0.8206	1341.7779 ± 714.4009
Ascending permutation	8.8971 ± 0.4559	129.0192 ± 16.4557	11.4387 ± 1.2143	682.2795 ± 186.3804	8.9463 ± 1.3739	137.7018 ± 43.3219	13.1311 ± 1.5016	967.5211 ± 509.6258
Median focus stitching	8.5286 ± 0.4087	107.9235 ± 9.4779	10.7801 ± 1.2695	603.4065 ± 193.3818	9.3805 ± 1.1299	154.1889 ± 57.7006	14.8729 ± 0.8407	1331.3416 ± 513.6267
Low-value centralized splicing	10.8415 ± 0.5097	183.8227 ± 24.2431	12.9614 ± 0.9211	928.1955 ± 218.6253	9.3665 ± 1.8376	179.0958 ± 80.3549	12.3807 ± 1.4782	814.0282 ± 614.1063

**Table 9:** Experimental results of multi-scale layers.

	FD001		FD002		FD003		FD004	
	RMSE	Score	RMSE	Score	RMSE	Score	RMSE	Score
3 layers	9.4289 ± 0.4064	127.3629 ± 8.3789	13.1086 ± 1.3404	1118.4028 ± 396.9302	9.2858 ± 0.5296	127.2645 ± 18.3399	16.2684 ± 1.3794	2187.0142 ± 959.6129
4 layers	9.0298 ± 0.3424	120.9700 ± 9.2074	11.6776 ± 0.7547	657.1125 ± 111.2070	9.5341 ± 0.5314	146.5535 ± 23.6192	13.4110 ± 1.0723	1362.7439 ± 521.0034
5 layers	8.5286 ± 0.4087	107.9235 ± 9.4779	10.7801 ± 1.2695	603.4065 ± 193.3818	9.3805 ± 1.1299	154.1889 ± 57.7006	14.8729 ± 0.8407	1331.3416 ± 513.6267
6 layers	8.4304 ± 0.5194	107.1597 ± 17.0268	10.9054 ± 0.7480	587.3027 ± 70.7027	9.4737 ± 1.0183	149.6954 ± 54.6117	14.4422 ± 0.6922	1507.5733 ± 539.6574

**Table 10:** Experimental results of low-frequency and high-frequency cross-scale interaction.

	FD001		FD002		FD003		FD004	
	RMSE	Score	RMSE	Score	RMSE	Score	RMSE	Score
All low frequencies	8.9206 ± 1.7994	119.8858 ± 59.8542	11.1570 ± 2.6408	755.4482 ± 23.6929	9.951 ± 1.4401	169.2580 ± 67.4891	18.0107 ± 1.5494	3097.838 ± 1552.7780
All high frequencies.	9.4356 ± 0.4644	140.8185 ± 17.1785	10.9371 ± 1.1324	611.1009 ± 251.8867	10.4167 ± 0.7831	182.8258 ± 44.7776	17.6666 ± 0.7357	2721.7449 ± 695.0262
No cross-scale	8.5522 ± 0.6289	111.7129 ± 16.7937	12.0940 ± 0.5883	624.3664 ± 53.8765	9.9520 ± 1.0563	170.3877 ± 53.8765	14.9102 ± 0.8254	1416.2726 ± 503.9176
Cross-scales	8.5286 ± 0.4087	107.9235 ± 9.4779	10.7801 ± 1.2695	603.4065 ± 193.3818	9.3805 ± 1.1299	154.1889 ± 57.7006	14.8729 ± 0.8407	1331.3416 ± 513.6267

**Table 11:** RMSE and score comparison of different methods.

Methods	FD001		FD002		FD003		FD004	
	RMSE	Score	RMSE	Score	RMSE	Score	RMSE	Score
DCNN&BiGRU [41]	12.99	198	16.10	1575	12.48	290	18.29	1741
IMDSSN [42]	12.14	206.11	17.40	1775.15	12.35	229.54	19.78	2852.81
SDFEPN [30]	11.14	163.93	14.51	807.23	12.73	229.93	16.34	1507
DBRNNs [26]	17.97	459.89	20.54	2064.39	19.94	658.12	23.97	2869.67
DCNN [43]	12.61	273.7	22.36	10412	12.64	284.1	23.31	12466
STAR [44]	10.61	169	13.47	784	10.71	202	15.87	1449
IDMFFN [30]	12.18 ± −0.43	204.69 ± 21.60	19.17 ± 0.26	1819.42 ± 289.59	11.89 ± 0.49	205.54 ± 23.37	21.72 ± 0.73	3382.84 ± 492.90
Proposed method	8.5286 ± 0.4087	107.9235 ± 9.4779	10.7801 ± 1.2695	603.4065 ± 193.3818	9.3805 ± 1.1299	154.1889 ± 57.7006	14.8729 ± 0.8407	1331.3416 ± 513.6267

**Table 12:** Comparison of ablation experiments of this model.

Dataset	Metrics	Proposed Method	Proposed Method/Feature Construction	Proposed Method/Cross-Scale Interaction	Proposed Method/Context Gating
FD001	RMSE	8.5286 ± 0.4087	47.333 ± 4.2018	8.5865 ± 0.7138	8.8799 ± 0.3491
	Score	107.9235 ± 9.4779	9992.7361 ± 5814.0089	112.5028 ± 18.4831	120.0873 ± 9.2578
FD002	RMSE	10.7801 ± 1.2695	61.5890 ± 7.0816	12.0948 ± 0.5883	11.2564 ± 0.7113
	Score	603.4065 ± 193.3818	799221.4528 ± 521295.8441	624.3664 ± 63.2752	615.214 ± 76.9968
FD003	RMSE	9.3805 ± 1.1299	33.4058 ± 5.4379	9.9520 ± 1.0563	9.4831 ± 1.3042
	Score	154.1889 ± 57.7006	5259.6456 ± 2353.5161	170.3877 ± 53.8765	157.0212 ± 66.9010
FD004	RMSE	14.8729 ± 0.8407	69.68 ± 9.7765	13.9102 ± 0.8254	13.9901 ± 0.9082
	Score	1331.3416 ± 513.6267	980086662.44 ± 127408228	1316.2727 ± 503.9176	1369.6185 ± 510.5561

## Data Availability

https://github.com/chongcc802/RUL/releases/download/v1.0/RUL.zip (accessed on 5 July 202).
